# Versatile Click Linker Enabling Native Peptide Release from Nanocarriers upon Redox Trigger

**DOI:** 10.1021/acs.bioconjchem.3c00484

**Published:** 2023-12-11

**Authors:** Erik R. Hebels, Stefanie Dietl, Matt Timmers, Jaimie Hak, Antionette van den Dikkenberg, Cristianne J.F. Rijcken, Wim E. Hennink, Rob M. J. Liskamp, Tina Vermonden

**Affiliations:** † Division of Pharmaceutics, Utrecht Institute for Pharmaceutical Sciences (UIPS), Utrecht University, Utrecht 3508 TB, The Netherlands; ‡ Cristal Therapeutics, Maastricht 6229 EV, The Netherlands; § Department of Biochemistry, Cardiovascular Research Institute Maastricht (CARIM), Maastricht University, Maastricht 6229 ER, The Netherlands; ∥ School of Chemistry, University of Glasgow, Glasgow G12 8QQ, U.K.

## Abstract

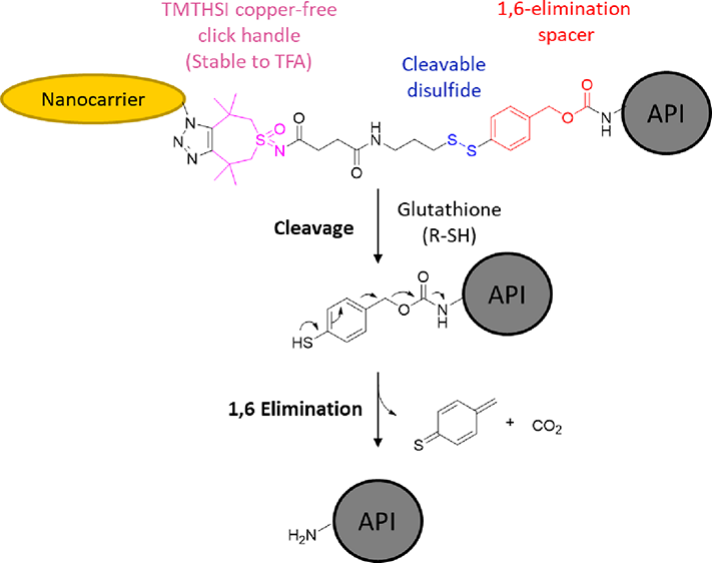

Nanocarriers have shown their ability to extend the circulation time of drugs, enhance tumor uptake, and tune drug release. Therapeutic peptides are a class of drug compounds in which nanocarrier-mediated delivery can potentially improve their therapeutic index. To this end, there is an urgent need for orthogonal covalent linker chemistry facilitating the straightforward on-the-resin peptide generation, nanocarrier conjugation, as well as the triggered release of the peptide in its native state. Here, we present a copper-free clickable ring-strained alkyne linker conjugated to the N-terminus of oncolytic peptide LTX-315 via standard solid-phase peptide synthesis (SPPS). The linker contains (1) a recently developed seven-membered ring-strained alkyne, 3,3,6,6-tetramethylthiacycloheptyne sulfoximine (TMTHSI), (2) a disulfide bond, which is sensitive to the reducing cytosolic and tumor environment, and (3) a thiobenzyl carbamate spacer enabling release of the native peptide upon cleavage of the disulfide via 1,6-elimination. We demonstrate convenient “clicking” of the hydrophilic linker–peptide conjugate to preformed pegylated core-cross-linked polymeric micelles (CCPMs) of 50 nm containing azides in the hydrophobic core under aqueous conditions at room temperature resulting in a loading capacity of 8 mass % of peptide to polymer (56% loading efficiency). This entrapment of hydrophilic cargo into/to a cross-linked hydrophobic core is a new and counterintuitive approach for this class of nanocarriers. The release of LTX-315 from the CCPMs was investigated in vitro and rapid release upon exposure to glutathione (within minutes) followed by slower 1,6-elimination (within an hour) resulted in the formation of the native peptide. Finally, cytotoxicity of LTX CCPMs as well as uptake of sulfocyanine 5-loaded CCPMs was investigated by cell culture, demonstrating successful tumor cell killing at concentrations similar to that of the free peptide treatment.

## Introduction

Nanocarriers loaded with drugs offer several advantages, including the protection of drugs from premature degradation, prolonged circulation time, selective tissue targeting, cellular internalization, and controlled drug release.^1−3^ To this end, covalent (temporary) entrapment of drugs improves stability of the platform during circulation.^4^ For novel active pharmaceutical ingredients (APIs) such as therapeutic peptides,^5,6^ there is a need for mild orthogonal chemistry to facilitate conjugation of such (prodrug) entities to nanocarrier vehicles.^7^ While employing a biologically relevant trigger, target-selective release should result in the native peptide, hence without any remaining conjugation fragment.^8^

**Scheme 1:**
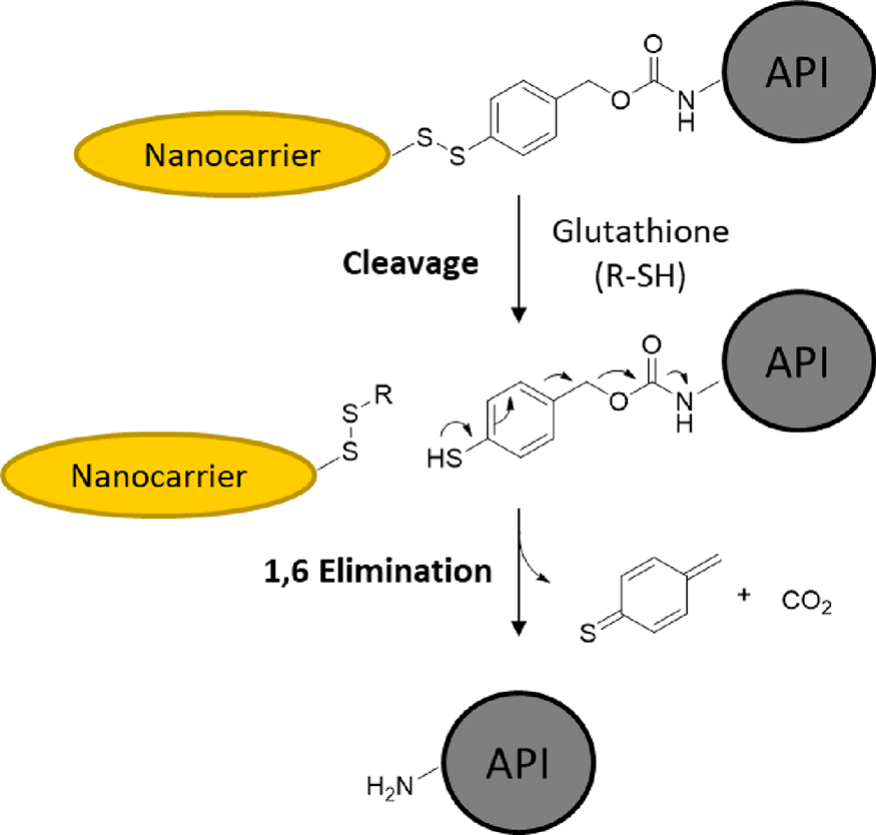
Glutathione-Mediated Cleavage and Subsequent 1,6-Elimination of the Thiobenzyl Carbamate Intermediate Resulting in the Release of the Native Amine Containing API^19^

Since the introduction of self-immolative connectors by Katzenellenbogen and co-workers in 1981,^9^ a plethora of examples for the triggered traceless release of APIs has been described. These include antibody–drug conjugates,^10^ polymer–drug conjugates,^11^ supramolecular hydrogel–drug conjugates,^12^ DNA–drug conjugates,^13^ liposome-loaded prodrugs,^14^ as well as carriers that themselves self-immolate.^15^ Reduction-sensitive linkages (RSLs) such as the disulfide bond are particularly interesting release triggers owing to the increased levels of glutathione (GSH) in the cytosol as well as the tumor environment (in the case of oncology applications) as compared to plasma and extracellular fluids.^16−18^ By utilizing the previously described 1,6-elimination chemistry of thiobenzyl carbamates,^19^ conveniently short RSL handles that rapidly dissociate upon disulfide cleavage can be synthesized (see Scheme 1). Examples for the native release of peptides and proteins from carriers and polymers upon exposure to mild reducing conditions have been described, further highlighting the potential of RSL release strategies.^20−22^

Although much work on self-immolative linker chemistry has been conducted on the releasability of cargos, less focus has been given to the convenient and efficient prodrug to carrier conjugation. Copper-free click chemistry presents an attractive approach owing to the mild, noncytotoxic, and generally orthogonal nature of this chemistry that even allows for coupling reactions in vivo.^23−25^ However, in the case of site specifically modified peptide–linker conjugates, the click handle has to withstand relatively harsh deprotection conditions, such as trifluoroacetic acid (TFA) treatment commonly employed during standard solid-phase peptide synthesis (SPPS).

Current readily available ring-strained alkynes such as dibenzocyclooctyne (DBCO, which is rather bulky and hydrophobic) and the later improved bicyclo[6.1.0]non-4-yn-9-ylmethanol (BCN, which is less bulky but still hydrophobic) have limited applications in SPPS since they do not endure the typical TFA deprotection conditions, requiring multistep procedures.^26^ A recently developed seven-membered ring-strained alkyne, 3,3,6,6-tetramethylthiacycloheptyne sulfoximine (TMTHSI), was demonstrated a more reactive and more hydrophilic click handle as compared to DBCO and BCN and, importantly, stable during TFA deprotection reactions.^27,28^ Furthermore, the higher hydrophilicity of the TMTHSI click handle allows for linker/conjugate applications in aqueous solutions, which is particularly beneficial for conjugation of peptides to nanoparticles dispersed in water.

In this work, we aimed to design and synthesize an amine reactive RSL linker based on thiobenzyl carbamate chemistry with TMTHSI as a reactive click handle for conjugation to an azide-containing carrier. By making use of the TFA resistance of TMTHSI, we site-specifically conjugate this linker to the N-terminus of LTX-315 (a multiamine containing oncolytic peptide under clinical investigation by intratumoral injection)^29−31^ via an on-the-resin approach using standard SPPS. To demonstrate straightforward conjugation of the linker–peptide conjugate to a nanocarrier, we selected clinically evaluated^4,32−36^ core-cross-linked polymeric micelles (CCPMs) based on partially methacrylated methoxy poly(ethylene glycol)-*b*-poly[*N*-(2-hydroxypropyl) methacrylamide-lactate]) (mPEG-*b*-pHPMAmLac_n_-MA) polymers that we partially modified with azidoacetic acid to introduce azide handles into the hydrophobic CCPM core. Finally, we investigated the release kinetics of LTX-315 under reducing conditions, as well as the cellular uptake and cytotoxicity of the platform in vitro to demonstrate the functionality, efficiency, and versatility of the designed linker.

## Results and Discussion

### Linker Synthesis

**Scheme 2:**
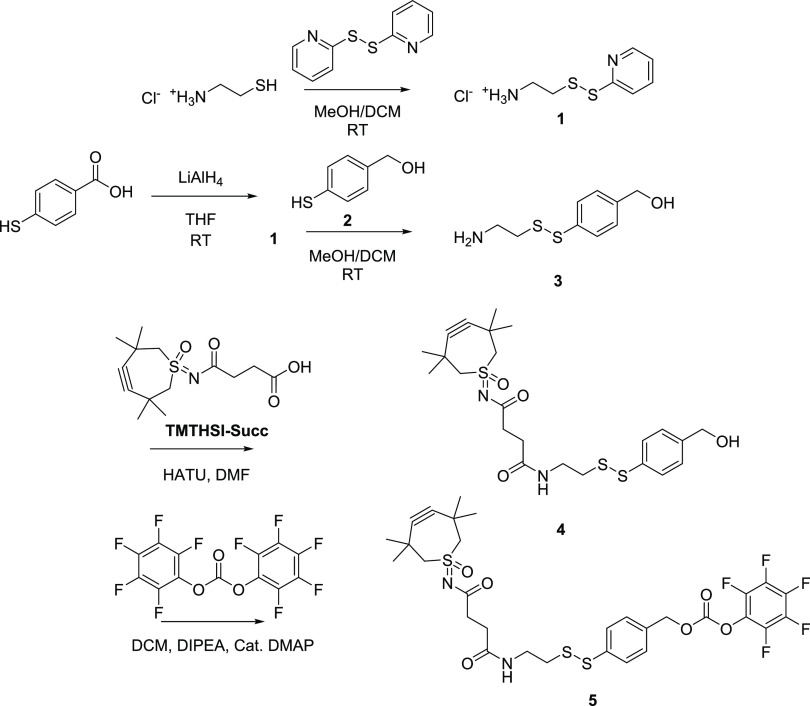
Synthesis Scheme of the Amine-Reactive, TMTHSI-Incorporated RSL Linker (Compound **5**)

We designed a linker to couple an amine-containing peptide to a nanoparticle and subsequently release the native peptide under reducing conditions, as found in the cytosol of cells and in tumor environments. This linker contains on one end an amine reactive pentafluorophenyl carbonate and on the other end TMTHSI as a clickable handle for conjugation to azide-containing nanoparticles, bridged by a spacer that self-immolates upon cleavage of the connecting disulfide (see Schemes 1 and 2). In doing so, this advances upon previously reported similar strategies employing RSL chemistries through incorporation of a robust click handle.^20,22^

Starting with cysteamine HCl, the thiopyridine-activated disulfide **1** was prepared via a known reaction with aldrithiol (2,2′-dithiodipyridine). Purification by repeated precipitation in diethyl ether (removing unreacted aldrithiol and the 2-thiopyridine byproduct) was efficient for obtaining a flaky white solid. The LiAlH_4_ reduction of 4-mercaptobenzoic acid yielded 4-mercaptobenzyl alcohol (**2**), following a previously described method.^20,37^ The reaction of compound **1** with **2** under nitrogen yielded the disulfide containing building block **3**. Purification of compound **3** was done by silica column chromatography. We proceeded with the coupling of TMTHSI-succinic acid to compound **3** present in the fraction (9:1 CH_2_Cl_2_:MeOH with 1–3% triethylamine), while ensuring a large excess (5-fold) of compound **3**. The identity of compound **4** was confirmed by TLC-MS (see Figure S2.4). The pentafluorophenyl activation of compound **4** to yield compound **5** (aimed linker) was carried out after a few washing steps for which DMAP was used as a catalyst. The identity of compound **5** was confirmed by NMR (Figure S1.3) and TLC-MS (Figure S2.5) attaining a final yield of 86% relative to TMTHSI-succ. ^19^F NMR analysis further confirmed that the intended pentafluorophenyl carbonate activation occurred, ruling out a potential dimerized carbonate ester (Figure S1.4). The purity (>95%) of compound **5** was established by HPLC (see Figure S3.1).

### Linker-LTX on Resin Conjugation

**Scheme 3:**
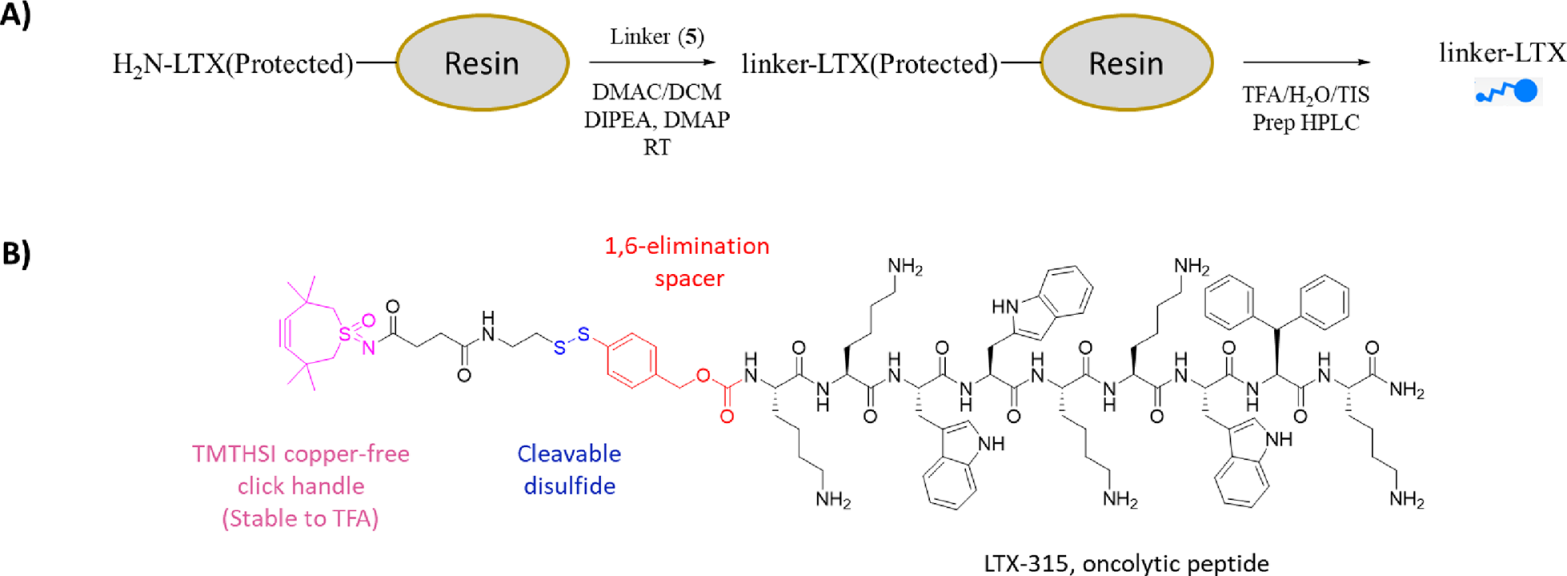
Overview of (A) the On-Resin Synthesis of the Linker-LTX Conjugate and (B) the Structure of the N-Terminal-Modified Linker-LTX-315 Conjugate

The stability of the TMTHSI click handle toward TFA deprotection conditions^27,28^ allows for on-the-resin conjugation of the linker and subsequent selective N-terminal modification of peptides (Fmoc deprotected N-terminus, protected side chains while on the resin). Particularly, a peptide such as LTX-315 (containing five lysine residues) benefits from this site-specific approach to obtain a well-defined and characterizable conjugate (Scheme 3). We employed a 1:1 equivalency ratio of linker to theoretical peptide content on resin to limit use of the click linker (compound **5**) reagent (up to 6-fold excess of reagents are employed in standard SPPS modifications^38^). Using DMAP as a catalyst, successful conjugation was indeed shown from disappearance of the linker from the reaction mixture as seen by TLC. The linker-LTX conjugate was obtained through preparative HPLC, and identity/high purity (1962 Da, >90%) was confirmed by MALDI-MS/HPLC (Figures S2.1 and S3.2). Unconjugated LTX-315 peptide (45 mol %) was also obtained after preparative HPLC, demonstrating that the linker was indeed the limiting reagent as intended and 9.4 mg (or 4.8 nmol) of linker-LTX conjugate and 5.6 mg (3.9 nmol) of unconjugated LTX-315 were isolated, corresponding with a conjugation yield of 55%.

### Polymer Derivatization

**Scheme 4:**
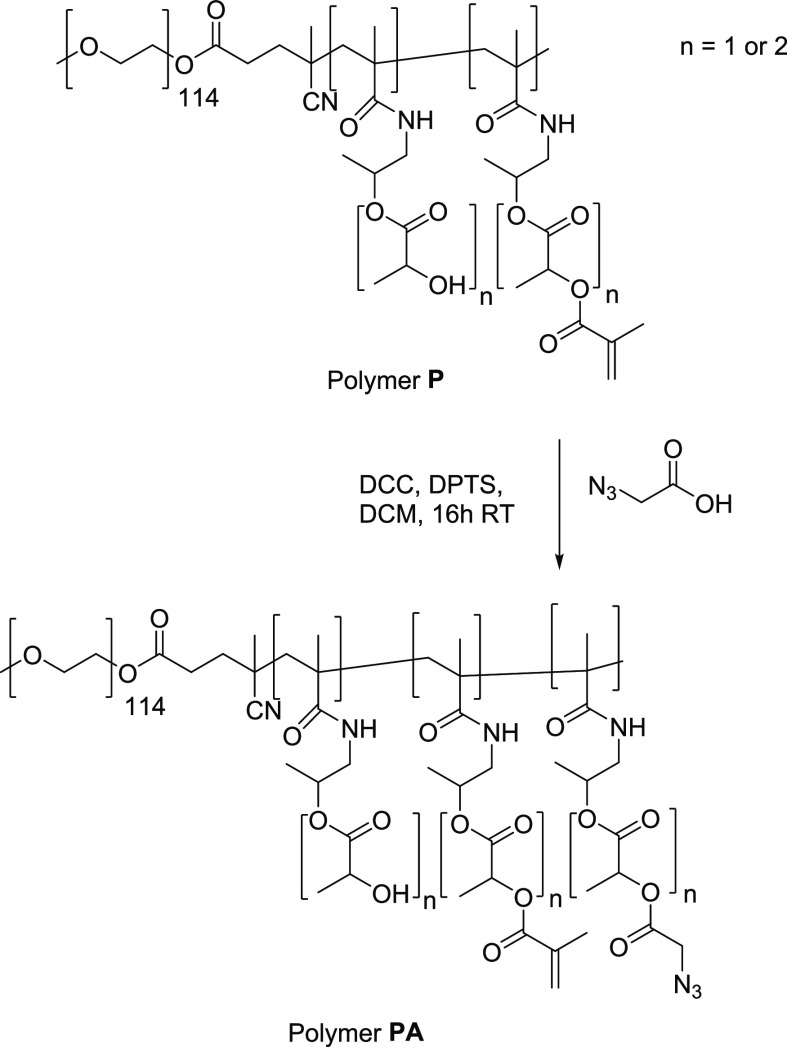
Modification of the mPEG-*b*-pHPMAmLac_*n*_-MA Polymer (**P**) with Azidoacetic Acid to Yield (**PA**) Polymer **P** has an mPEG block of 5 kDa and based on NMR analysis, and there are an average of approximately 23 and 28 units of HPMAmLac_1_ and HPMAmLac_2_ per polymer chain, respectively. Additionally, there are approximately seven methacrylate functionalities per polymer chain. The total *M*_n_ is thus approximately 20 kDa. The extent of azide derivatization in polymer **PA** is reported in Table 1.

We selected a previously described polymeric micellar system based on thermosensitive methacrylated methoxy poly(ethylene glycol)-*b*-poly[*N*-(2-hydroxypropyl) methacrylamide-lactate]) (mPEG-*b*-pHPMAmLac_*n*_-MA) (abbreviated as polymer **P,** see Figure S1.5)^32^ for coupling of the linker-LTX conjugate. Polymer **P** is used as a building block for CCPMs through free-radical polymerization above its cloud point (CP, which depends on copolymer composition of the thermosensitive block) in aqueous buffer. Eight percent of the available hydroxyl moieties on the side chains were modified with methacrylate moieties (enabling cross-linking, resulting in a polymer with a CP of 11 °C). The remaining 92% of the hydroxyl side groups of polymer **P** are available for additional modification, and azides were introduced via a Steglich esterification^39^ with azidoacetic acid (AAA) to obtain polymer **PA** (see Scheme 4). AAA was selected as it is the smallest existing azide-containing carboxylic acid (to minimize possible impact on the polymer characteristics such as CP, see Table 1).

**Table 1:** Characteristics of Azide-Functionalized mPEG-*b*-pHPMAmLac_*n*_-MA Polymers

	azides per polymer chain							
polymer	feed^a^	obtained^b^	*M*_n_ (kDa)^c^	*M*_n_ (kDa)^d^	**Đ**^d^	cloud point (°C)^e^	micelle diameter (nm)^f^	micelle PDI^f^
**P**			19.6	22.3	1.68	11	58	0.04
**PA5**	2.6	1.3	19.0	23.3	1.76	8	55	0.05
**PA10**	5.2	3.3	18.8	24.0	1.95	5	53	0.02
**PA15**	7.7	5.3	19.7	24.6	2.03	1	48	0.03

a Feed is expressed in units per polymer chain (mol/mol).

b Determined by NaOH hydrolysis followed by ultrahigh-performance liquid chromatography (UHPLC) analysis.

c Determined by ^1^H NMR analysis.

d Determined by gel permeation chromatography (GPC) analysis using PEG calibration standards for the average number molecular weight (*M*_n_) and polydispersity (*Đ*) of the polymer.

e Determined by onset of light scattering at a 90° angle of the polymer dissolved in phosphate buffer (100 mM Na_2_HPO_4_, adjusted to pH 7.3 using HCl).

f Determined by dynamic light scattering (DLS) analysis of noncross-linked polymers at 25 °C in phosphate buffer (100 mM Na_2_HPO_4_, adjusted to pH 7.3 using HCl).

Successful azide modification in a feed ratio-dependent manner was confirmed by IR spectroscopy (Figure S5.1) and quantification achieved through NaOH hydrolysis of the polymer followed by UHPLC analysis of reformed AAA. The coupling efficiency ranged from 50 to 70%. Increased azidoacetic acid derivatization in **PA** resulted in a linear decrease in CP as well as hydrodynamic diameter of noncross-linked micelles measured at 25 °C, due to the increased hydrophobicity resulting in stronger dehydration of the core.^40^

### CCPM Entrapment and Release

**Figure 1:**
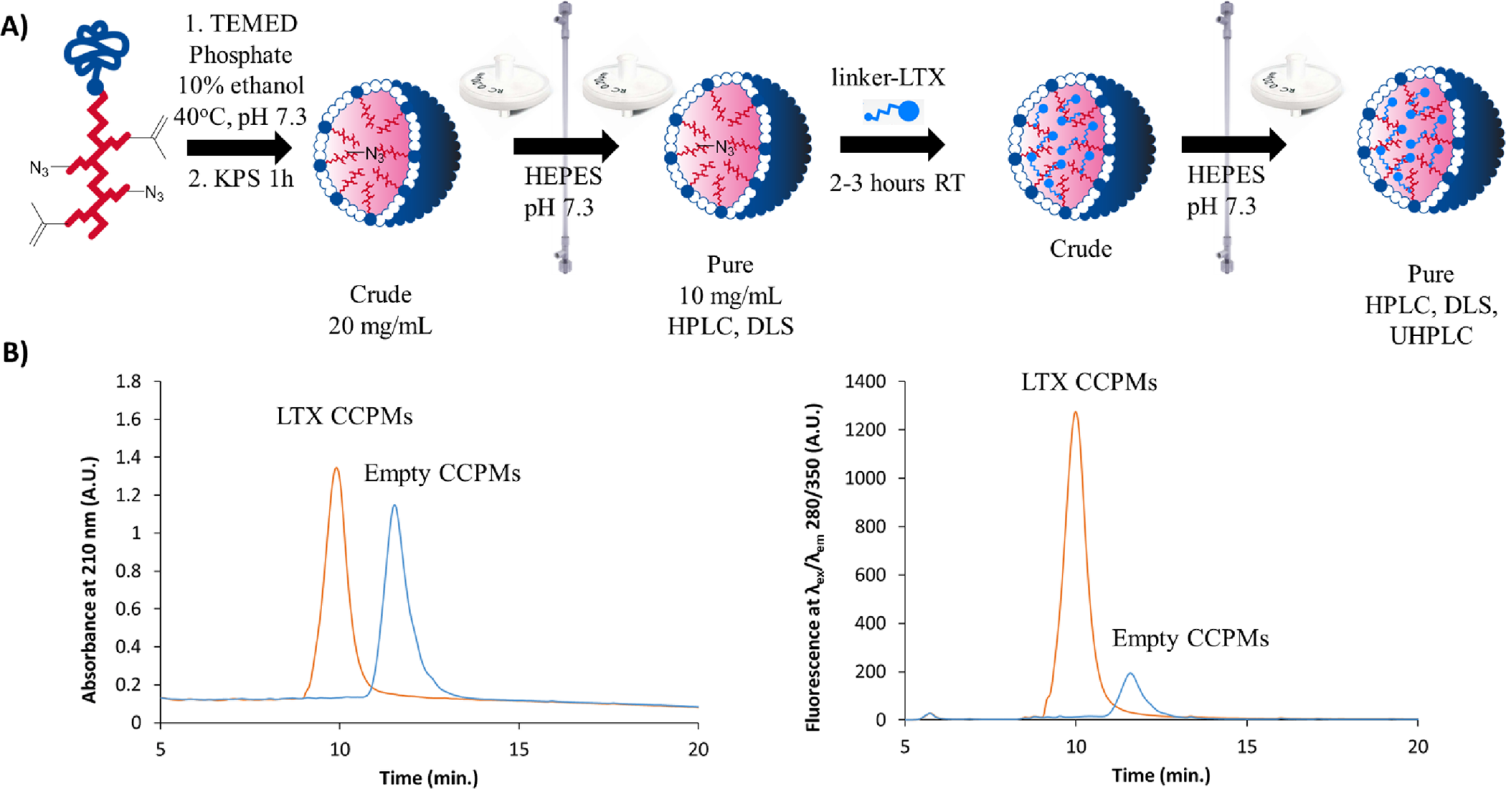
(A) Overview of the synthesis of CCPMs and subsequent covalent coupling of the linker-LTX conjugate, including filtration, TFF purification, and analytical steps and (B) HPLC chromatograms of the LTX-loaded CCPMs after TFF purification (orange), compared to reference empty CCPMs (blue), recorded with 210 nm absorbance (left) and λ_ex_/λ_em_ 280/350 nm fluorescence (right). The small signal of empty CCPMs in the right chromatogram is attributed to light scattering.

CCPMs of PA15 were obtained by free-radical polymerization of the methacrylated side chains after micellization at 40 °C (well above the CP of 1 °C) in phosphate buffer (pH 7.3) with 10% ethanol, following a previously described procedure (Figure 1A).^32^ This core-cross-linking reaction has to be conducted before introduction of the linker-LTX conjugate (in contrast to mixing before cross-linking^40,41^), as larger peptides (particularly those containing lysine, methionine, and tryptophan residues) are prone to side reactions under the conditions employed here.^42,43^ Following tangential flow filtration (TFF) purification of the CCPMs, a diameter of 57 nm with a PDI of <0.1 was measured by DLS, slightly larger than the noncross-linked micelles based on the same polymers (48 nm). This increase in diameter has been observed previously in analogous systems^32^ and is attributed to the presence of 10% ethanol, which swells the core at the moment of cross-linking, the latter being initiated by addition of potassium persulfate (KPS). TFF purification was carried out using HEPES buffer as salt exchange as phosphate salts caused precipitation of the linker-LTX conjugate, as previously reported for other lysine-containing peptides.^44^ The polymer content after TFF purification, as determined by lactic acid content with UHPLC after NaOH hydrolysis, was 43 mg, indicating losses of around 30% using this purification method (polymer feed during cross-linking was 60 mg). Additionally, AAA content was found to be lowered to 3.2 azides per polymer chain (compared to 5.3 azides per polymer chain of the noncross-linked polymer), likely caused by aggregation of higher hydrophobic azide-containing CCPMs.

We clicked the linker-LTX conjugate, dissolved in water, into the CCPM obtained after TFF (in 100 mM HEPES buffer, pH 7.3) by simply mixing the peptide solution and micellar dispersion at room temperature for 3 h (Figure 1A). An excess amount of azides relative to the linker-LTX conjugate (2.6 fold) was employed with the CCPMs to promote efficient entrapment. The degree of the peptide coupling was determined by analyzing the micelles using HPLC and hallmarked by a shift in retention time and the presence of a LTX-associated tryptophan fluorescence signal at λ_ex_/λ_em_ 280/350 nm^45^ of the CCPMs as compared to empty CCPMs (Figure 1B). A substantial decrease (>95%, based on absorbance) in the linker-LTX peak was recorded prior to TFF purification (Figure S3.3), demonstrating that the LTX conjugate had almost quantitatively reacted, as anticipated.

The LTX CCPMs had a Z-Ave of 59 nm with a PDI of 0.07 as determined by DLS, indicating that the CCPMs retained their size after loading (57 nm for empty CCPMs) as would be expected from a cross-linking-before-loading approach. The polymer content of LTX-loaded CCPMs was 38.4 mg, which corresponds to a slight loss of 10% that occurred during the final TFF purification. It is noted that the volume after TFF purification was adjusted to the starting volume.

Given the hydrophilicity of LTX-315 and the linker-LTX conjugate (exemplified by the short retention time on HPLC, Figures S3.2 and S3.3 as well as the excellent solubility in water), it is counterintuitive that this linker-LTX conjugate can penetrate into the hydrophobic CCPM core and subsequently undergo the click reaction.^46,47^ The robust click reaction is anticipated to be the driving force and traps the conjugate upon entering or encountering the core. Additionally, azides located on the outermost parts of the hydrophobic core are likely responsible for the click conjugation, since an excess amount of azides was employed, embedding LTX within the hydrophilic PEG layer. The 5 kDa PEG shell thickness of micelles has previously been reported to be in the range of 5–10 nm,^48,49^ which could fully shield even the unlikely extended conformation of LTX-315, having a contour length of 3.2 nm (assuming 0.35 nm per amino acid).^50^ In buffer of pH 7.3, both empty CCPMs and LTX CCPMs had a neutral zeta potential (−2.1 ± 8.9 and −0.2 ± 9.6 mV, respectively) demonstrating PEG shielding of the loaded LTX-315 with protonated lysine residues indeed occurred. Finally, we also entrapped the fluorescent dye S.Cy5-DBCO (nonreducible conjugation for imaging purposes) in CCPMs. A >95% entrapment was achieved based on absorbance (see Figure S3.4), further highlighting the high efficiency of a click-entrapment of a hydrophilic compound in the hydrophobic core.

**Figure 2:**
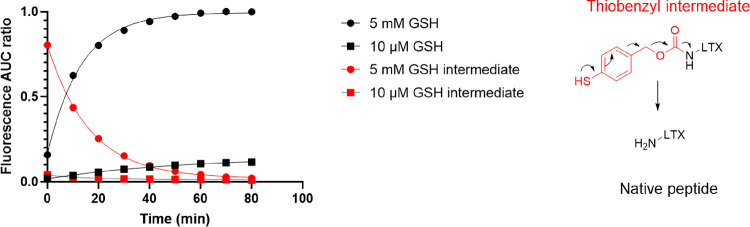
Release kinetics of LTX-315 from LTX CCPMs under reducing conditions in the cytosol (5 mM GSH, circles) versus typical plasma concentration (10 μM GSH, squares). The thiobenzyl carbamate intermediate that undergoes 1,6-elimination to yield native LTX-315 was detected as well (squares). See Figure S4.1 for an illustration of obtained chromatograms. Recorded with λ_ex_/λ_em_ 280/350 nm fluorescence, expressed as the AUC ratio to maximal recorded fluorescence.

We investigated the release of LTX from the purified CCPMs at glutathione (GSH) concentrations resembling those found in the cytosol of living cells (5 mM) and blood (10 μM)^16−18,20^ by UHPLC (Figure 2). The release of LTX from the micelles in HEPES with 10 μM GSH and at 37 °C was slow (less than 10% during incubation for 1.5 h). However, upon incubation of the CCPMs in HEPES with 5 mM GSH and the same temperature, rapid cleavage of the disulfide bond was observed, releasing the thiobenzyl intermediate that then subsequently underwent 1,6-elimination yielding the native peptide, LTX-315, reaching a plateau within 60 min (see Figure S4.1 for illustration of chromatograms). Initial rapid disulfide cleavage was also previously reported for aliphatic disulfides in hydrophilic nanogels (for the release of ovalbumin in the presence of GSH). The observed rapid disulfide cleavage in our study suggests that the linker-LTX conjugate likely resides within the hydrophilic PEG corona rather than in the less accessible hydrophobic core.^51^ The identity of released/native LTX-315 was further confirmed by MALDI-MS (Figure S2.3).

The releasable LTX content from the CCPMs upon exposure to 5 mM GSH was quantified with a calibration-free LTX-315. Figures 2 and S4.1 show that the released amount of LTX reached a plateau after around 60 min incubation. The maximum released amount corresponds with 8% mass loading of LTX-315 with respect to polymer content. This in turn points to 56% entrapment efficiency of the total LTX-315 feed, which was determined by GSH treatment of the reaction sample before purification. Entrapment losses can be attributed (other than conjugation efficiency) to losses during TFF purification, reaction of linker-LTX conjugate with traces of uncoupled azido acetic acid (Figure S2.6 showing the presence of uncoupled AAA-linker-LTX), disproportionation of the linker-LTX conjugate (Figure S2.2 showing the presence of a dimer LTX-SS-LTX), and finally side-product formation resulting in incomplete 1,6-elimination as previously described for dithiobenzyl carbamates.^22^ Importantly, the potential side products and impurities are efficiently removed by TFF purification, as shown in Figure S3.5.

### Cellular Internalization and Toxicity

**Figure 3:**
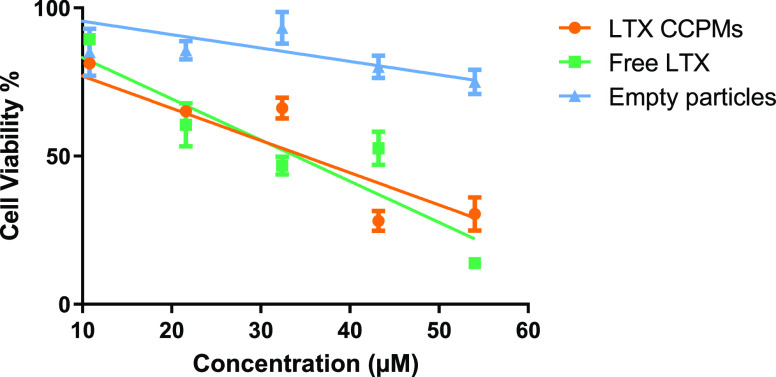
Cell viability of HeLa cells after incubation with LTX CCPMs (orange), free LTX-315 peptide (green), or empty CCPMs (blue) for 24 h at 37 °C, as determined by MTS assays. Error bars represent the standard error of mean of 3 wells.

We investigated the cytotoxicity of LTX CCPMs on HeLa cancer cells using an MTS assay (Figure 3). The HeLa cells incubated with LTX CCPMs showed a significantly reduced cell viability. On the other hand, incubation of the cells with empty CCPMs diluted to match the concentration of the LTX-loaded CCPMs (1.4 mg/mL CCPM content as the highest) showed a slight decrease in cell viability, demonstrating minimal toxicity of the nanocarrier itself, in line with previous reports.^52,53^ LTX-315 is a cationic membrane penetrating lytic peptide that in its free form is taken up by cells to exert its effects (also highlighted by the cytotoxicity of free LTX in Figure 3).^29^ Therefore, the CCPMs could either be internalized by the cells and release their cargo intracellularly or release cargo extracellularly in the culture environment.

**Figure 4:**
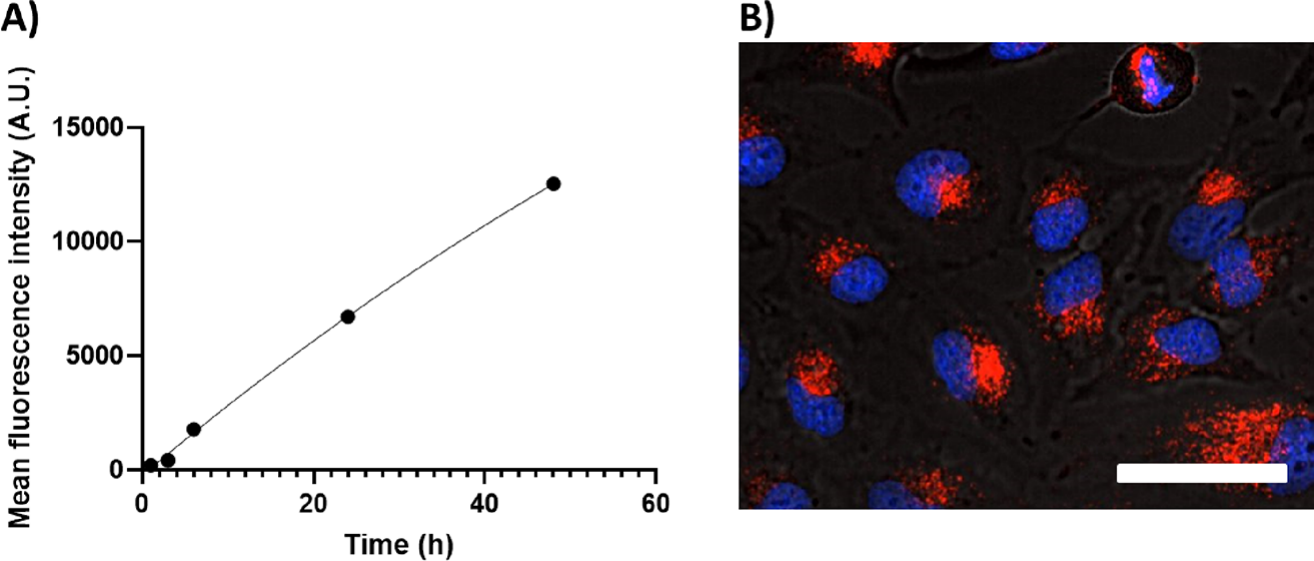
HeLa cell uptake of S.Cy5-labeled CCPMs (not loaded with LTX) at a concentration of 1.4 mg/mL represented by (A) transition of mean cell fluorescence intensity as determined by flow cytometry and (B) overlaid laser confocal scanning microscopy image after 24 h incubation at 37 °C with S.Cy5-loaded CCPMs showing brightfield, nuclear stain (blue) and S.Cy5-loaded CCPMs (red). Scale bar set to 25 μm.

The cellular internalization of CCPMs was studied by using S.Cy5-labeled CCPMs (Figure 4). A time-dependent increase in intracellular fluorescence was observed, with accumulation in regions outside the nucleus (likely within endosomal compartments) in a similar fashion as reported previously for proapoptotic peptide nanoparticles.^20^ These results indicate that CCPM uptake and subsequent intracellular release play a role, but do not fully explain the observed cytotoxicity (after 24 h, not all CCPMs are taken up, yet similar toxicity to free LTX-315 is found). It is also unlikely that the CCPMs release the peptide in sufficient amounts in the nonreducing cell culture medium at the start of the incubation with the intact cells. However, the equally potent cytotoxic effect of free and CCPM-entrapped LTX is likely due to complete release of LTX from all CCPMs in the culture medium following initial cell lysis, exposing reducing agents such as GSH. Additional studies on stability in plasma as well as sterically hindered disulfide iterations of the linker would be of interest to explore in future studies.^54^

## Conclusions

A versatile click-based linker chemistry for the covalent entrapment and reduction triggered release of native therapeutic peptides from nanocarriers was developed. The linker described here is an example of a straightforward N-terminal site-specific peptide derivatization via solid-phase chemistry to generate a clickable peptide construct. This work also showcases peptide–linker entrapment into already generated CCPMs. This way, cross-linking chemistry such as free-radical polymerization can be employed for particle formation without the risk of damaging the therapeutic cargo. Furthermore, a high loading capacity and entrapment efficiency are attained with this click approach and rapid native peptide release results upon exposure to cytosolic/tumor relevant reducing conditions. Finally, any other compound with an amine could be considered for conjugation with this linker, multilysine peptides being particularly challenging owing to the presence of multiple amines. This opens application toward other amine containing APIs and other azide-containing nanocarriers or drug delivery systems.

## Materials and Methods

### Materials

The copper-free click handle 3,3,6,6-tetramethylthiacycloheptyne sulfoximine (TMTHSI) conjugated with succinic acid (TMTHSI-succ) as well as methacrylated methoxy poly(ethylene glycol)-*b*-poly[*N*-(2-hydroxypropyl) methacrylamide-lactate]) (mPEG-*b*-pHPMAmLac_*n*_-MA) with an *M*_n_ of 20 kDa was provided by Cristal Therapeutics (Maastricht, The Netherlands) and synthesized as described previously.^27,32^ Dibenzocyclooctyne-functionalized sulfonated cyanine 5 (Sulfo.Cy5-DBCO) was obtained from Lumiprobe (Hannover, Germany). All other materials were obtained from Sigma-Aldrich (Zwijndrecht, The Netherlands) unless indicated otherwise. All solvents were obtained from Biosolve (Valkenswaard, The Netherlands).

### Synthesis

#### Linker Synthesis

##### Synthesis Compound**1**

In a typical reaction, aldrithiol-2 (3.9 g, 17.6 mmol) was dissolved in 10 mL of dichloromethane (DCM)/MeOH (1/1 v/v) to which cysteamine hydrochloride (1.0 g, 8.8 mmol) dissolved in 20 mL of MeOH was added dropwise while bubbling with nitrogen. After 16 h of stirring at RT, the mixture was dropped in 1 L of cold diethyl ether while stirring to precipitate the formed compound **1** and left to stir for 30 min followed by filtration through a sintered glass filter. The obtained yellow solid was redissolved in 20 mL of MeOH, and 1 L of diethyl ether was subsequently added to the same flask to precipitate again. Then, the isolated solid was redissolved in 20 mL of methanol, precipitated in 200 mL of diethyl ether, centrifuged, and dried under nitrogen, yielding 1.5 g (74%) of pyridine dithioethylamine hydrochloride, a flaky white solid. ^1^H NMR (400 MHz, D_2_O): δ 8.31 (ddt, *J* = 5.0, 1.7, 0.8 Hz, 1H), 7.69 (tdd, *J* = 7.4, 1.8, 0.7 Hz, 1H), 7.61 (dq, *J* = 8.1, 0.9 Hz, 1H), 7.19 (ddt, *J* = 7.5, 5.0, 0.9 Hz, 1H), 3.20 (t, 2H), 2.97 (t, *J* = 6.3 Hz, 2H). See Figure S1.1 for the ^1^H NMR spectrum.

##### Synthesis Compound **2**

LiAlH_4_ (1.5 g, 39.0 mmol) was suspended in 30 mL of dry THF while stirring in an ice bath, and 4-mercaptobenzoic acid (2 g, 13.0 mmol) dissolved in 30 mL of dry THF was added dropwise. After addition, the ice bath was removed and the reaction mixture was left to stir overnight at RT. The reaction was quenched with 3 mL of Milli-Q water in an ice bath, and 100 mL of 2 N HCl was added to acidify the reaction mixture. Compound **2** was then extracted three times with 100 mL of EtOAc, washed with 100 mL of Milli-Q and 100 mL of brine, dried over anhydrous sodium sulfate, and concentrated. The obtained off-white semisolid was purified by silica chromatography employing 2:1 hexane:EtOAc as an eluent (*R*_f_ = 0.3), yielding 1.5 g (82%) of 4-mercaptobenzyl alcohol as a white solid. ^1^H NMR (400 MHz, CDCl_3_) δ 7.28–7.20 (m, 4H), 4.62 (s, 2H). See Figure S1.2 for the ^1^H NMR spectrum.

##### Synthesis Compound**3**

Compound **2** (0.71 g, 3.2 mmol) was dissolved in 10 mL of MeOH, and compound **1** (0.60 g, 3.4 mmol) was dissolved in 20 mL of DCM and was added dropwise with bubbling nitrogen while stirring. After 30 min, the reaction mixture was diluted with DCM and applied onto a silica column employing 9:1 DCM:MeOH until elution of the yellow mercaptopyridine side product, followed by 9:1 DCM:MeOH with 1–3% triethylamine to elute compound **3** (*R*_f_ = 0.3). No concentrating was done to prevent disproportionation reactions, the next reaction being conducted in the elution solvent.

##### Synthesis Compound**4**

A successive reaction step was conducted directly using compound **3** in the elution solvent (approximately 250 mL 9:1 DCM:MeOH, 1–3% triethylamine). TMTHSI-succinic acid (0.21 g, 0.68 mmol) was weighed together with hexafluorophosphate azabenzotriazole tetramethyl uronium (HATU) (0.29 g, 0.75 mmol), dissolved in 10 mL of DMF, and added to the excess compound **3** (4-fold based on the starting reagents used for compound **3**). After stirring for 30 min, the reaction mixture was washed three times with 100 mL of 0.1 N HCl, once with saturated NaHCO_3_, once with brine, dried over sodium sulfate, and filtered. The product was not concentrated or further isolated. The identity of compound **4** was confirmed by TLC-MS: expected mass for C_23_H_32_N_2_O_4_S_3_Na^+^ is 519.1, found 518.8.

##### Synthesis Compound **5**

In the final step, bis(pentafluorophenyl) carbonate (0.54 g, 1.37 mmol) was added to the washed compound **4** mixture, followed by *N*,*N*-diisopropylethylamine (DIPEA) (1.2 mL, 6.8 mmol) and a catalytic amount of 4-dimethylaminopyridine (DMAP). The reaction was monitored by TLC. After 1–2 h, the reaction mixture was washed three times with 100 mL of 0.1 N HCl, two times with saturated NaHCO_3,_ once with brine, dried over sodium sulfate, and concentrated to obtain a brown oil. The oil was redissolved in EtOAc and run on a silica column employing 1:1 hexane:EtOAc until elution of unreacted bis(pentafluorophenyl) carbonate, followed by only EtOAc (*R*_f_ = 0.5). After concentrating, 0.41 g of gooey off-white solid was obtained with a yield of 85% with respect to TMTHSI-succ. ^1^H NMR (400 MHz, DMSO-d6): δ 8.01 (t, *J* = 5.7 Hz, 1H), 7.61 (d, *J* = 8.3 Hz, 2H), 7.50 (d, *J* = 8.6 Hz, 2H), 5.40 (s, 2H), 3.90 (d, *J* = 13.9 Hz, 2H), 3.69 (d, *J* = 13.9 Hz, 2H), 3.37–3.24 (m, 2H), 2.84 (t, *J* = 6.3 Hz, 2H), 2.42 (t, *J* = 7.2 Hz, 2H), 2.27 (t, *J* = 7.2 Hz, 2H), 1.35 (s, 6H), 1.18 (s, 6H). ^19^F NMR (565 MHz, DMSO-d6): δ −154.11 (d, *J* = 19.5 Hz, 2F), −157.26 (t, *J* = 23.1 Hz, 1F), −162.11 to −162.22 (m, 2F). TLC-MS: Expected mass for C_30_H_31_N_2_O_6_S_3_F_5_Na^+^ is 729.1, and found 729.8. See Figure S3.1 for the HPLC chromatogram of purified compound **5**.

#### Peptide Synthesis

Synthesis of LTX-315 (KKWWKKW-Dip-K-NH_2_) was performed by microwave solid-phase peptide synthesis (SPPS) in a Liberty Blue peptide synthesizer (CEM Corp., Matthews, NC, USA) following a standard Fmoc/tBu protocol on a 0.1 mmol scale. A high-swelling Tentagel XV rink amide resin (loading 0.2–0.4 mmol/g) (Rapp Polymere, Tuebingen, Germany) was employed as a solid support. Standard couplings of amino acids were performed at 0.2 M in dimethylformamide (DMF) using DIC/OxymaPure activation, with the first lysine loaded in a double coupling procedure (the coupling method used was optimized to the corresponding amino acid according to the recommended operation of Liberty Blue). Fmoc removal was carried out using 20% piperidine in DMF. Modification of LTX-315 with a click linker (compound **5**) was carried out before cleavage/final deprotection from the solid support as described in detail in the next paragraph.

#### Linker-LTX Conjugate Synthesis

The coupling of the click linker (compound **5**) with LTX-315 was performed on the resin to attain selective N-terminal conjugation. About 0.05 mmol of resin content (half batch from the SPPS) was suspended in 5 mL of dimethylacetamide (DMAC)/DCM (1:1 v/v) followed by addition of DIPEA (170 μL, 1.0 mmol). Compound **5** was dissolved in DCM to a 50 mg/mL stock solution and added to the resin (700 μL, 35 mg, 0.05 mmol) followed by a catalytic amount of DMAP (1 mg, 8.2 μmol). After stirring for 1.5 h at RT, the resin was washed three times with 10 mL of DMF/DCM (1:1 v/v) and three times with 10 mL of DCM and subsequently dried under nitrogen flow. The peptide conjugate was then cleaved/deprotected by stirring for 1 h using 5 mL of trifluoroacetic acid (TFA)/triisopropylsilane (TIS)/water, 95:2.5:2.5 (v/v/v). Following filtration, the crude peptide was precipitated in 45 mL of cold diethyl ether, centrifuged, washed with diethyl ether, dried under nitrogen flow, dissolved in Milli-Q water:acetonitrile (ACN) 1:1, and lyophilized.

The lyophilized crude peptide–linker conjugate (35 mg) was dissolved in 4 mL of 5% ACN in Milli-Q water supplemented with 0.1% formic acid and filtered using a 0.45 μm recombined cellulose syringe filter. Fractionation was carried out using preparative reverse-phase high performance liquid chromatography (Prep-RP-HPLC) on a Waters 2535 quaternary gradient module with a Waters 2489 UV/Visible detector (detection at 210 and 280 nm) and ReproSil-Pur 120 C18-AQ (10 μm, 25 mm × 250 mm, Dr. Maisch) column. ACN/water supplemented with 0.1% formic acid was used as an eluent at a flow of 25 mL/min and a gradient of 5–95% ACN over 60 min. Fractions were manually collected and analyzed by HPLC (Figure S3.2) and MALDI-MS (Figure S2.1), and pure fractions were combined and lyophilized (see Figure S3.3 for HPLC of the pooled linker-LTX conjugate).

#### Polymer Derivatization with Azides

Azide functionalities were introduced onto the thermosensitive block of (mPEG-*b*-pHPMAmLac_*n*_-MA, polymer **P,** characteristics are reported in Figure S1.5) as shown in Scheme 2, a detailed description of the synthesis of this polymer is described by Hu et al.^32^ Stock solutions of azidoacetic acid (AAA) (50 mg/mL), DPTS (20 mg/mL), and DCC (50 mg/mL) in dry DCM were prepared. The mPEG-*b*-pHPMAmLac_*n*_-MA polymer (1.0 g, 50 μmol, based on *M*_n_ of 20 kDa determined by NMR, containing 2.6 mmol of free −OH groups) was dissolved in dry DCM (final concentration 100 mg/mL), followed by addition of AAA with a feed ratio of 5.5 mol % (14.5 mg, 143 μmol), 11.0 mol % (29.0 mg, 287 μmol), and 16.5 mol % (43.5 mg, 430 μmol) relative to HPMAmLac_*n*_–OH groups, 0.1 equiv to AAA) of DPTS and finally 1.1 equiv (to AAA) of DCC. The reaction mixtures were stirred at RT for 16 h. The mixture was then filtered using a 0.2 μm PTFE syringe filter, and the polymers were precipitated three times in diethyl ether and dried under vacuum overnight. The azide modification was confirmed by IR spectroscopy (Figure S5.1). The obtained polymers were further characterized for composition, molecular weight, and CP as described in the Characterization section. The synthesized polymers with 5.5, 11.0, and 16.5% AAA feed are referred to as **PA5**, **PA10,** and **PA15**, respectively.

#### Core-Cross-Linked Polymeric Micelle (CCPM) Formation

CCPM formation was carried out following a previously published procedure.^32^ In detail, an ice cold solution of polymer **PA15** (4.15 mL, 24.1 mg/mL) was stirred and purged with N_2_ for 15 min and mixed with tetramethylethylenediamine (TEMED, 125 μL, 120 mg/mL), both dissolved in phosphate buffer (100 mM Na_2_HPO_4_, adjusted to pH 7.3 using HCl). The mixture was warmed to 40 °C, and after 10 min, 0.5 mL of ethanol was added (following the previously described procedure^32^) dropwise to the now opalescent mixture. After 15 min, potassium persulfate (KPS, 338 μL, 30 mg/mL, dissolved in phosphate buffer (100 mM Na_2_HPO_4_, adjusted to pH 7.3 using HCl) was added to the micellar dispersion while under N_2_ flow. After 1 h, the CCPM dispersion was filtered through a 0.2 μm RC syringe filter and purified by tangential flow filtration (TFF) against HEPES buffer (10 mM HEPES, adjusted to pH 7.3 using NaOH) at a polymer concentration of 10 mg/mL, employing an mPES membrane (50 kDa cut off, 20 cm^2^, MicroKros filter modules, Repligen) for 40–50 washing volumes. Following TFF, 1 mL of 1 M HEPES buffer was added resulting in ∼100 mM HEPES salt content with pH 7.3, and the dispersion was filtered again with a 0.2 μm RC syringe filter. The polymer concentration of the obtained dispersion was determination as described in the Characterization section.

#### CCPM Loading

The lyophilized linker-LTX conjugate was dissolved in Milli-Q (6.0 mg, 2.7 μmol) to 20 mg/mL, added to 6 mL of TFF purified CCPMs (∼7 mg/mL, 7.2 μmol total azide content, determined by hydrolysis followed by UHPLC as described in the Characterization section), and stirred for 3 h at room temperature. An excess of azides (2.6 fold) was employed to promote quantitative coupling of the linker-LTX conjugate. Purification was carried out by TFF as described above, however using 100 mM HEPES and only 10 washing volumes to reduce polymer losses at this stage (see Scheme 4 for a complete overview).

CCPMs loaded with fluorescent sulfonated cyanine 5 (S.Cy5) for cell uptake studies were prepared by mixing 100 μL of S.Cy5-DBCO (dissolved to 10 mg/mL in DMSO, 1.0 μmol) with 5 mL of TFF purified CCPMs (∼7 mg/mL, 6.0 μmol total azide content) and stirring for 3 h at room temperature. Purification was carried out with TFF as described above.

### Characterization

#### Nuclear Magnetic Resonance (NMR) Spectroscopy

^1^H NMR spectra were recorded on an Agilent 400-MR NMR spectrometer (400 MHz; Agilent Technologies, Santa Clara, USA). Residual solvent peaks of CDCl_3_ (δ = 7.26 ppm), D_2_O (δ = 4.79), or D_6_ DMSO (δ = 2.50 ppm) were used to calibrate chemical shifts. ^19^F NMR spectra were recorded on a Bruker Avance Neo spectrometer (565 MHz).

#### Infrared Spectroscopy (IR)

IR spectra were recorded with solid polymer samples using an ATRU equipped Spectrum 2 (PerkinElmer, Llantrisant, UK) and reported as normalized transmission in cm^–1^.

#### Thin-Layer Chromatography (TLC)

TLC was performed by using aluminum-bound silica plates obtained from Merck Darmstadt (SiO_2_, Kieselgel 60 F254). Compounds were visualized by UV detection at 254 nm and, where applicable, stained with ninhydrin in DCM to visualize amines or potassium permanganate in 10% NaOH to visualize alkynes.

#### Mass Spectrometry (MS)

TLC-MS spectra were recorded on an expression high-performance compact mass spectrometer equipped with a Plate Express TLC plate reader (Advion, Ithaca, USA).

Matrix-assisted laser desorption ionization (MALDI)-MS spectra were recorded on an Ultraflextreme (Bruker Daltonics, Bremen, Germany), loading 1 μL of sample solution with 1 μL of matrix (10 mg/mL α-cyano-4-hydroxycinnamic acid in 30% ACN with 0.1% TFA).

#### High-Performance Liquid Chromatography (HPLC)

HPLC analysis was performed using an Alliance 2695 chromatography system with an XBridge C18 column (5 μm, 4.6 mm × 150 mm, Waters) at a column temperature of 25 °C and employing an Alliance 2487 ultraviolet (UV) detector at 210 nm and Alliance 2475 fluorescence (FL) detector at λ_ex_/λ_em_ 280/350 nm. Acetonitrile/water supplemented with 0.1% formic acid was used as an eluent at a flow of 1 mL/min and a gradient of 5 to 95% ACN over 20 min.

#### Gel Permeation Chromatography (GPC)

GPC for polymer analysis was performed using an Alliance 2695 (Waters) chromatography system with two PLgel 5 μm mixed-D columns (Polymer Laboratories) in series at a column temperature of 65 °C and employing a refractive index detector. DMF supplemented with 10 mM LiCl was employed as the eluent with an elution rate of 1 mL/min. Sample concentration was typically 10 mg/mL, and PEGs of narrow and defined molecular weights obtained from PSS (Germany) were used as calibration standards. Recording of data and calculations of molecular weights were done with Waters Empower 32 software.

#### Cloud Point (CP) Measurements

Using an adapted procedure,^55^ the CPs of the different thermosensitive polymers in phosphate buffer (100 mM Na_2_HPO_4_, adjusted to pH 7.3 using HCl, 5 mg/mL polymer) were determined by measurement of light scattering at a 90° angle upon the onset of opalescence. Scattered light intensity was measured using a Jasco FP-8300 spectrophotometer employing a wavelength of 550 nm with 1 nm slit width and a response time of 1 s. Temperature was ramped from 2 to 50 °C at 1 °C per minute.

#### Dynamic Light Scattering (DLS)

The size of the CCPMs was determined by DLS using a Malvern Zetasizer nano series ZS90 at a measurement angle of 90°. Measurements were carried out at 25 °C. Unless stated otherwise, the concentration of the micellar dispersions was approximately 10 mg/mL in either phosphate buffer (100 mM Na_2_HPO_4_, adjusted to pH 7.3 using HCl) or HEPES buffer (100 mM HEPES, adjusted to pH 7.3 using NaOH). The ζ-potential was determined with a Zetasizer Nano Z (Malvern ALV CGS-3, Malvern, UK) after 1000× dilution in 10 mM HEPES buffer at pH 7.4 (settings: temperature 25 °C, viscosity 0.8872 cP, RI 1.330, dielectric constant 78.5).

#### Ultrahigh-Performance Liquid Chromatography (UHPLC)

UHPLC analysis for the quantification of azidoacetic acid and lactic acid was performed using an Acquity (Waters) chromatography system with an HSS T3 column (1.7 μm, 2.1 mm × 100 mm, Waters) at a column temperature of 50 °C, sample temperature of 20 °C and employing an Acquity PDA detector at 210 nm. KH_2_PO_4_ buffer (10 mM, pH = 2.5) was used as an isocratic eluent at a flow of 0.5 mL/min for 2.5 min followed by an increasing gradient of acetonitrile supplemented with 0.1% phosphoric acid from 0 to 90% over 3 min.

UHPLC analysis for LTX-315 release quantification was performed using an Acquity (Waters) chromatography system with a CSH C18 column (1.7 μm, 2.1 × 50 mm, Waters) at a column temperature of 50 °C and sample temperature of 20 °C and employing an Acquity FL detector at λ_ex_/λ_em_ 280/350 nm. Acetonitrile/water supplemented with 0.1% formic acid was used as an eluent at a flow of 0.5 mL/min and a gradient of 5–95% ACN over 10 min.

#### Polymer Concentration Determination

The polymer concentration of the CCPM dispersions was determined through lactic acid concentration by UHPLC after hydrolysis as reported previously.^41^ Briefly, 20 μL of the CCPM dispersion was incubated with 10 μL of NaOH (1 M) for at least 2 h at 37 °C followed by the addition of 20 μL of HCl (1 M). Samples were run on UHPLC as described above. Sodium lactate was employed as the reference standard to determine the lactic acid concentration.

The polymer amount was calculated as follows: amount of polymer = measured amount of lactic acid × (*M* + 5000)/[90.08 × (*m* + 2*n*)], where *M* is the *M*_n_ of the thermosensitive block P(HPMAmLac_*n*_), *m* and *n* are the numbers of repeat units of HPMAmLac_1_ and HPMAmLac_2_ in the block copolymer (**P**), respectively (determined by ^1^H NMR, 24.8 and 26.8 units, respectively).

#### Azide Content Quantification by NaOH Hydrolysis

The extent of AAA functionalization of the **PA5**, **PA10**, and **PA15** polymers as well as **PA15** CCPMs was determined following a forced hydrolysis protocol and subsequent UHPLC analysis. The polymers were dissolved at 20 mg/mL in phosphate buffer (100 mM Na_2_HPO_4_, adjusted to pH 7.3 using HCl) of which 20 μL was mixed with 10 μL 1 M NaOH and incubated for 3 h at 37 °C. Then, 20 μL of 1 M HCl was added, and the samples were measured by UHPLC as described above. Calibrations of AAA were run with a range of 100–1000 μg/mL and the amount of azides per polymer chain calculated (moles of AAA divided by moles of polymer).

#### Release of LTX-315 from CCPMs

To 180 μL of purified LTX-loaded CCPMs was added either 20 μL of 50 mM or 100 μM GSH (obtained from Sigma-Aldrich, reduced form) dissolved in HEPES (100 mM, adjusted to pH 7.3 using NaOH) resulting in a GSH concentration of 5 mM and 10 μM, respectively, and the samples were analyzed by UHPLC with 5 μL injections at a 10 min interval for 80 min. Area under the curve (AUC) of the cleaved intermediate and native peptide was determined by integration using the empower software (Waters), and the AUC ratios were calculated relative to the highest AUC. For the release quantification, calibrations of free LTX-315 with a range of 100–500 μg/mL were generated and a sample treated with 5 mM GSH for 90 min was quantified.

### Cytotoxicity and Internalization Studies

#### Cell Culture

HeLa cells were cultured and maintained at 37 °C with Dulbecco’s modified eagle medium (DMEM) supplemented with 10% fetal bovine serum (FBS) in an incubator regulated with 5% CO_2_, 95% air, and saturated humidity. Cells were passaged every 2–4 days upon reaching 80% confluency using trypsin ethylenediaminetetraacetic acid (trypsin-EDTA).

#### Cell Viability

HeLa cells were plated into black polystyrene 96-well plates (Agilent #204626–100) at a density of 1.0 × 10^4^ cells per well and incubated for 24 h at 37 °C. The medium was aspirated, and 100 μL dilutions (dilutions made in 100 mM HEPES buffer, 5x dilution of sample into medium)) of LTX-loaded CCPMs, empty CCPMs, and free LTX-315 in DMEM medium supplemented with 10% FBS and with 1% penicillin/streptomycin were added in triplicate. After 24 h, 20 μL of MTS staining solution (CellTiter 96 AQ_ueous_, Promega) was added. Following a 2 h incubation, absorbance (490 nm) was recorded using a Mithras plate reader. Data were background subtracted and normalized using medium only wells and untreated cells of the same plate.

#### Cellular Uptake

##### Confocal Microscopy

HeLa cells with a cell density of 5000 cells/well were plated into a black polystyrene 96-well plate (Agilent #204626-100) in DMEM medium with 10% FBS and supplemented with 1% penicillin/streptomycin. The following day, HEPES dilutions of S.Cy5-loaded CCPMs (5× diluted in medium) were added to the cells in triplicate and incubated for 24 h at 37 °C. Prior to confocal microscopy, the cells were treated with 2 μg/mL Hoechst 33342 for 10 min in an incubator at 37 °C, 5% CO_2._ The cells were imaged in OptiMEM on a Yokogawa CV 7000 Microscope (40× water immersion objective lens). The Cy5 fluorescence was measured by excitation at 638 nm and emission at 676/29 nm and the fluorescence of the Hoechst 33342 by excitation 405 nm and emission at 445/45 nm.

##### Flow Cytometry

HeLa cells were plated into 96-well plates with a cell density of 5.000 cells per well and incubated for 1, 3, 6, 24, and 48 h at 37 °C, 5% CO_2_. Dilutions of S.Cy5-loaded CCPMs purified by TFF (dilutions made in 100 mM HEPES buffer, 5× dilution of sample into medium) in DMEM medium supplemented with 10% FBS and 1% penicillin/streptomycin were prepared, and after aspiration of culture medium, 100 μL of CCPM dispersion was added to the cells in triplicate. After incubation, the treatment medium was removed and the cells were washed with PBS. The cells were then harvested using 50 μL of trypsin followed by an incubation of 3 min at 37 °C, 5% CO_2_ to detach the cells. The cells were resuspended into culture medium and washed with PBS. The cells were fixated with 1% paraformaldehyde for 15 min and washed three times with 1% BSA solution. Finally, the cell-associated fluorescence was detected using a FACSCanto II flow cytometer (BD canto II) with 5 × 10^4^ cells per sample. The Cy5 fluorescence was measured by excitation at 633 nm and emission at 660/20 nm.

## Supporting Information Available

^1^H NMR data of compounds **1**, **2**, and **5** (linker, including ^19^F NMR) and polymer **P**; MADLI-MS spectra of the purified linker-LTX conjugate, the linker-LTX conjugate after freeze-drying, LTX-loaded CCPMs after treatment with 5 mM GSH and an HPLC fraction with *R*_t_ = 9.0 min that emerged during click entrapment of linker-LTX into the CCPMs; TLC-ESI-MS of compounds **4** and **5**; HPLC chromatograms of compound **5**, purified linker-LTX, click entrapment of Linker-LTX into CCPMs, click entrapment of S.Cy5-DBCO into CCPMs, and their subsequent purification; UPLC chromatograms of LTX-315 release kinetics from LTX-loaded CCPMs treated with 5 mM GSH; IR spectra of AAA-modified polymers (**PA**) (PDF)

## Abbreviations

AAA: azidoacetic acid
ACN: acetonitrile
AUC: area under curve
CCPM: core-cross-linked polymeric micelle
CP: cloud point
DCC: *N,N′-*dicyclohexylcarbodiimide
DCM: dichloromethane
DIPEA: *N*,*N*-diisopropylethylamine
DLS: dynamic light scattering
DMAP: 4-dimethylaminopyridine
DMEM: Dulbecco’s modified eagle medium
DMF: dimethylformamide
DMSO: dimethyl sulfoxide
DPTS: *N,N*-dimethylaminopyridinium *p*-toluenesulfonate
FBS: fetal bovine serum
GPC: gel permeation chromatography
HATU: hexafluorophosphate azabenzotriazole tetramethyl uranium
HeLa: cervical cancer cells isolated from Henrietta Lacks
HPMAmLac_*n*_: *N*-2-hydroxypropyl methacrylamide mono/di lactate
HPLC: high-performance liquid chromatography
IR: infrared
KPS: potassium persulfate
MA: methacrylic acid
MALDI: matrix-assisted laser desorption ionization
mPEG: methoxy polyethylene glycol
MS: mass spectrometry
MTS: 3-(4,5-dimethylthiazol-2-yl)-5-(3-carboxymethoxyphenyl)-2-(4-sulfophenyl)-2*H*-tetrazolium
NMR: nuclear magnetic resonance spectroscopy
**P**: mPEG-*b*-pHPMAmLac_*n*_-MA
**PA**: mPEG-*b*-pHPMAmLac_*n*_-MA-AAA
PM: polymeric micelle
RT: room temperature
TEMED: tetramethylethylenediamine
TFF: tangential flow filtration
THF: tetrahydrofuran
TLC: thin-layer chromatography
TMTHSI: 1-imino-3,3,6,6-tetramethyl-4,5-didehydro-2,3,6,7-tetrahydro-1*H*-1λ6-thiepine 1-oxide
TMTHSI-Succ: TMTHSI conjugated with succinic acid
UHPLC: ultrahigh-performance liquid chromatography
