# Supplementary material for: Versatile Click Linker Enabling Native Peptide Release from Nanocarriers upon Redox Trigger
Source: Bioconjug Chem. 2023 Dec 11;34(12):2375–86. doi: 10.1021/acs.bioconjchem.3c00484 (PMC10739580; doi:10.1021/acs.bioconjchem.3c00484)
Supplement: Supplementary file 1 — bc3c00484_si_001.pdf [file bc3c00484_si_001.pdf]

Supporting Information (SI)

Versatile click linker enabling native peptide release from nanocarriers upon redox trigger

Erik R. Hebels[1], Stefanie Dietl[1], Matt Timmers[1,2], Jaimie Hak[1], Antionette van den Dikkenberg[1], Cristianne J. F. Rijcken[2], Wim E. Hennink[1], Rob M. J. Liskamp[2,3,4], and Tina Vermonden[1]

[1] Division of Pharmaceutics, Utrecht Institute for Pharmaceutical Sciences (UIPS), Utrecht University, 3508 TB Utrecht, the Netherlands.

[2] Cristal Therapeutics, 6229 EV Maastricht, the Netherlands

[3] Department of Biochemistry, Cardiovascular Research Institute Maastricht (CARIM), Maastricht University, Maastricht 6229 ER, the Netherlands

[4] School of Chemistry, University of Glasgow, Glasgow G12 8QQ, U.K.

1. NMR Spectra

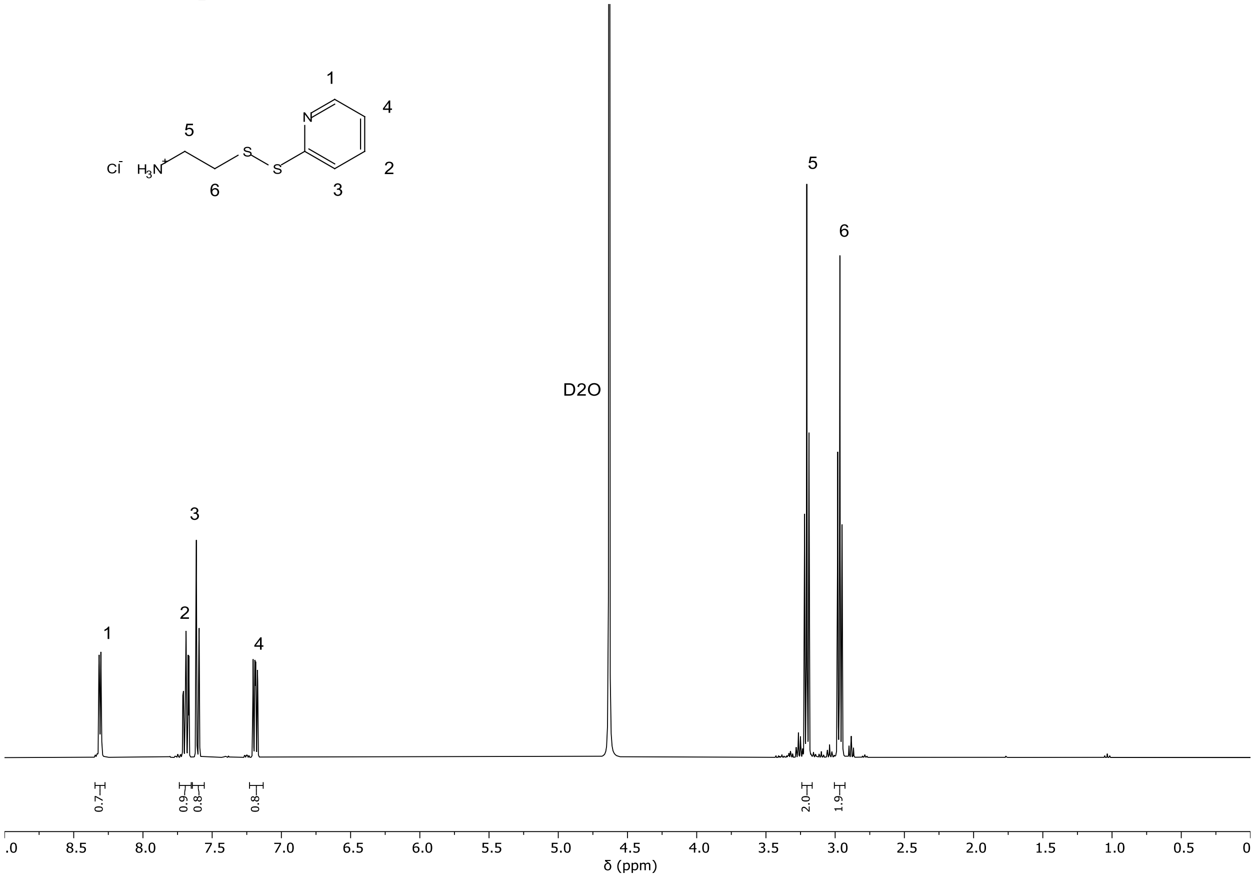

Figure S1.1: ^{1}H NMR spectrum of compound ***1*** *(pyridine dithioethylamine hydrochloride). The solvent was* D_2O.

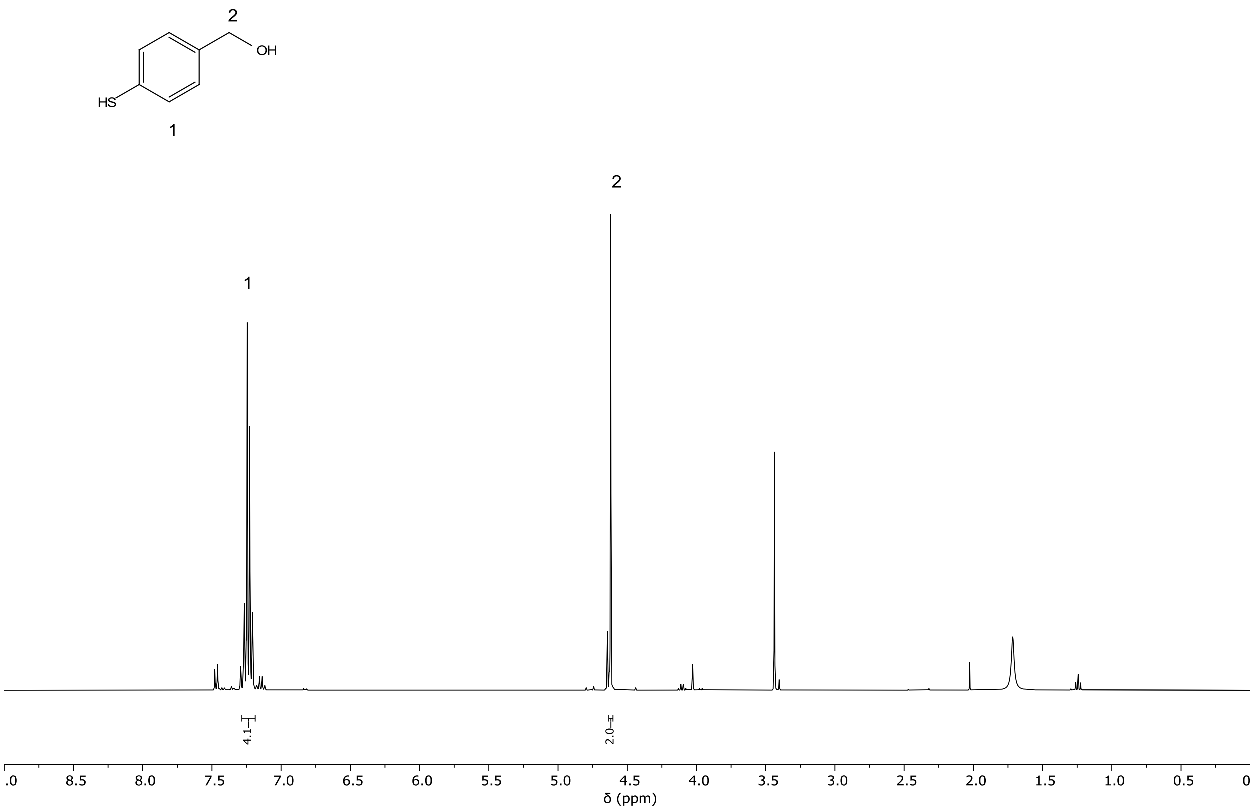

Figure S1.2: ^{1}H NMR spectrum of compound ***2*** *(4-mercaptobenzyl alcohol). The solvent was* $CDCl_3$.

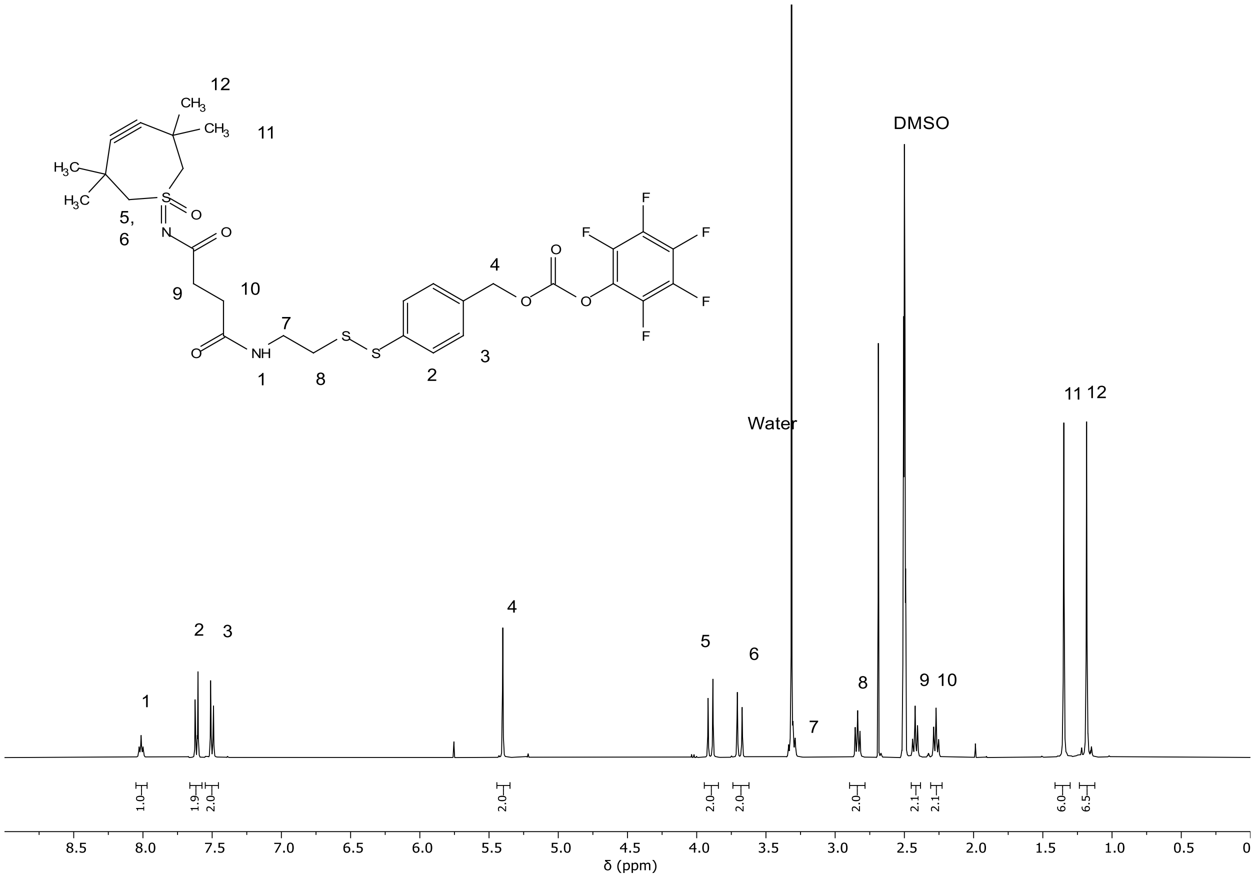

*Figure S1.3: ^{1}H NMR spectrum of the linker, compound **5**. The solvent was DMSO-d6.*

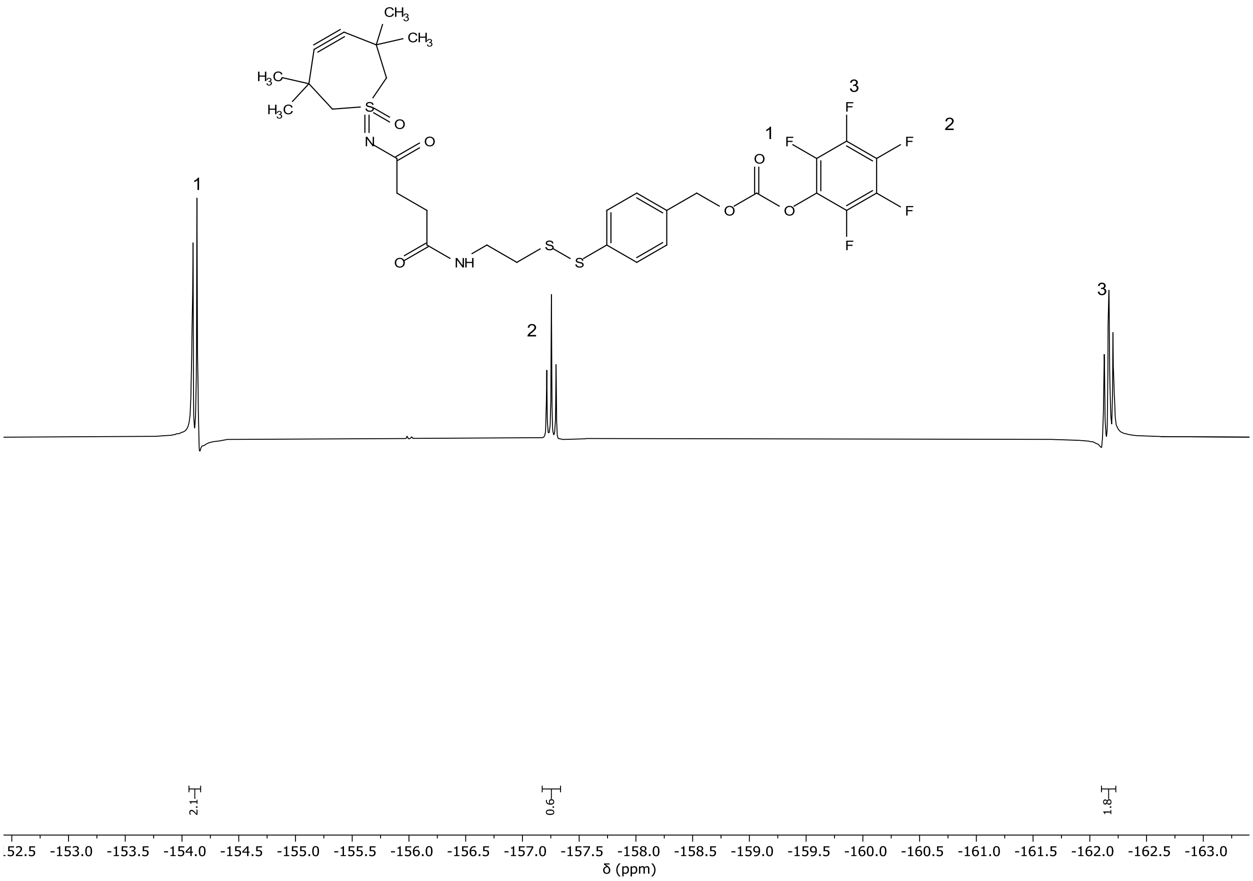

*Figure S1.4: ^{19}F NMR spectrum of compound **5**. The solvent was DMSO-d6.*

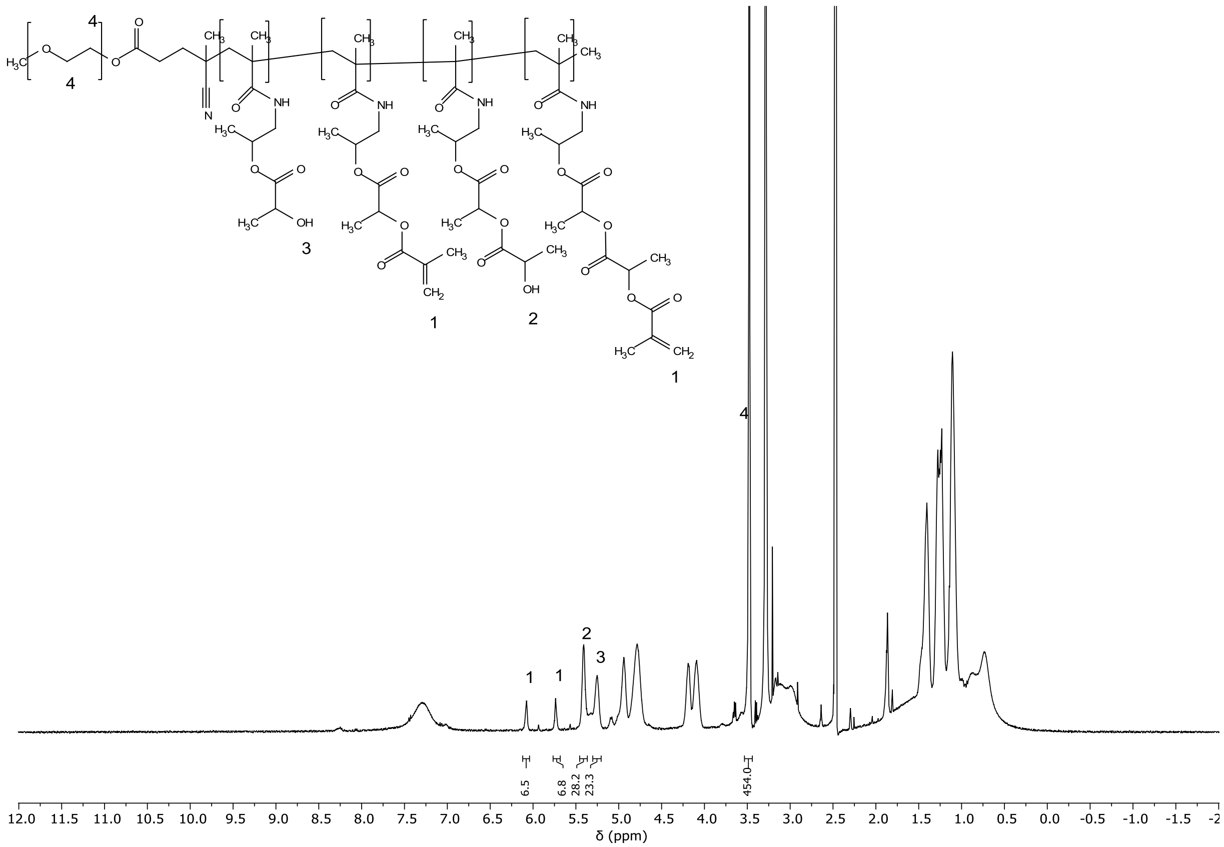

Figure S1.5: ^{1}H NMR spectrum of mPEG-b-pHPMAmLac$_n$-MA, polymer **P** has an mPEG molecular weight of 5 kDa and based on NMR analysis, there are approximately 23 and 28 units of HPMAmLac$_1$ and HPMAmLac$_2$ per polymer chain, respectively. Additionally, there are approximately 7 methacrylate functionalities per polymer chain. The total M_n is thus approximately 20 kDa. The solvent was DMSO-d6.

2. Mass Spectra

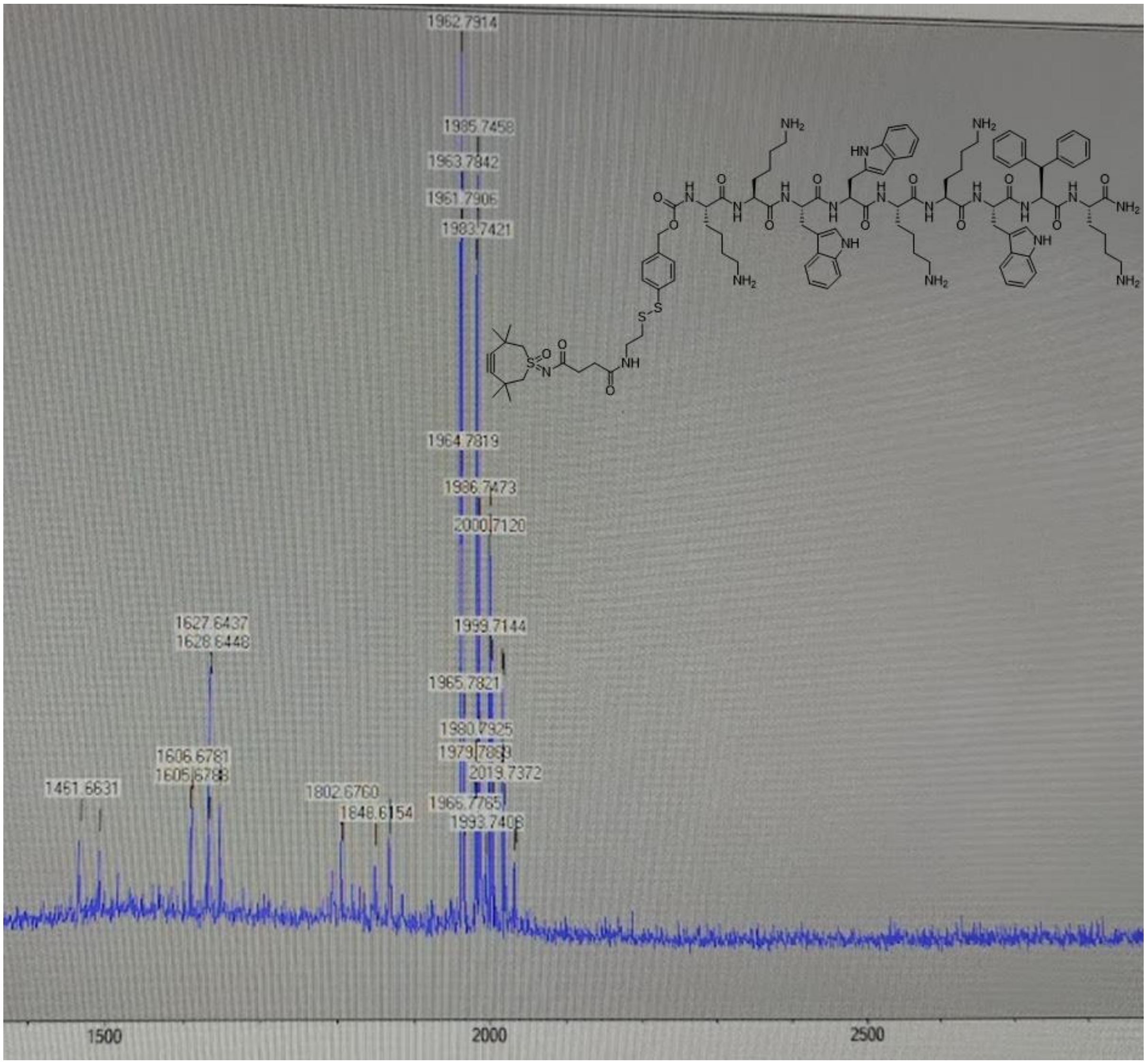

Figure S2.1: MALDI-MS spectrum of the preparative HPLC purified Linker-LTX conjugate (structure shown). Expected mass for $C_{102}H_{137}N_{20}O_{14}S_3^+$ is 1962.0, measured 1961.8, 1983.7 (Na^+ adduct) and 1999.7 (K^+ adduct).

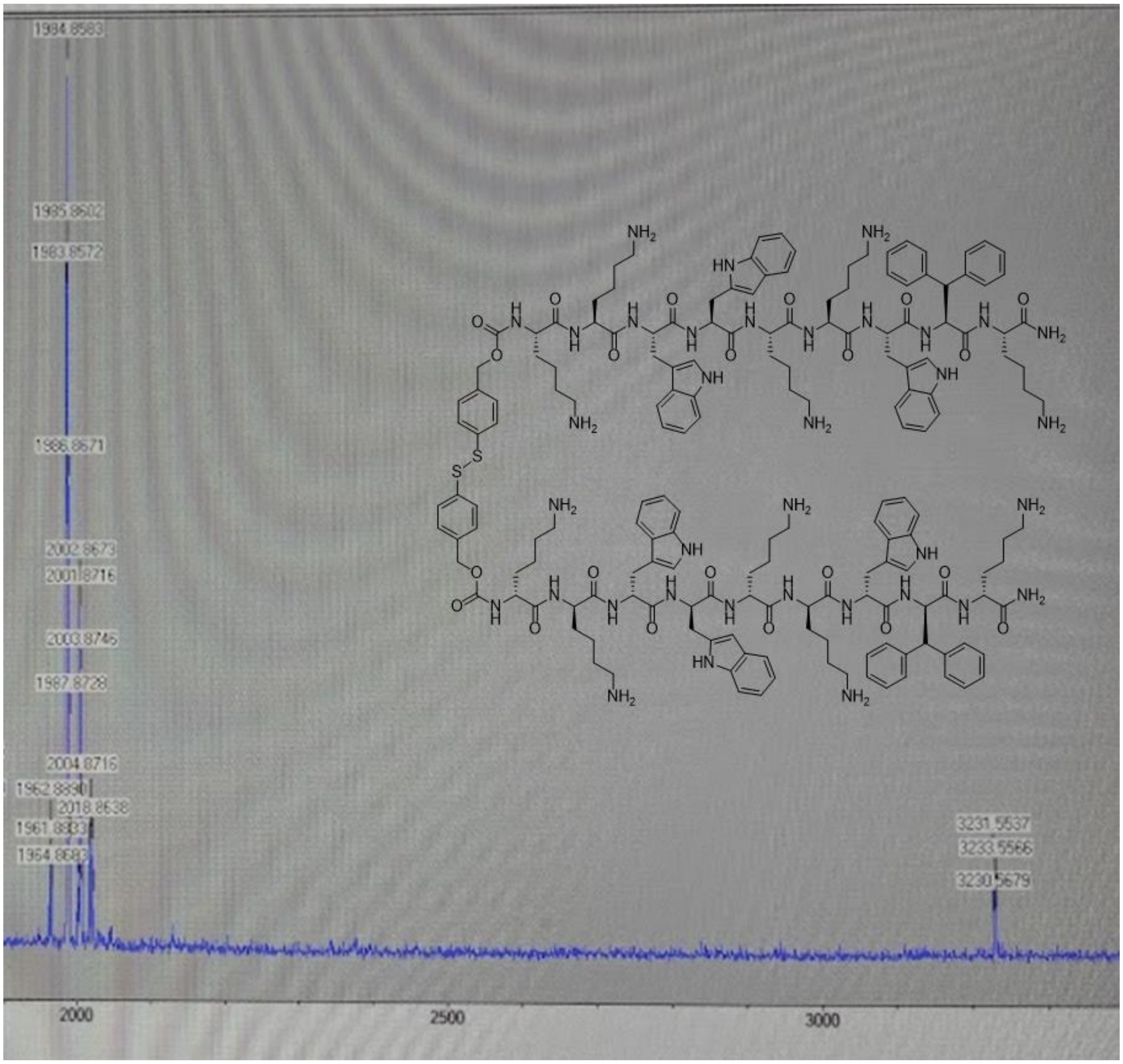

Figure S2.2: MALDI-MS spectrum of the preparative HPLC purified Linker-LTX conjugate after freeze drying and dissolution in milliQ. Expected mass for $C_{102}H_{137}N_{20}O_{14}S_3^+$ and $C_{102}H_{136}N_{20}O_{14}S_3Na^+$ is 1962.0 and 1984.0, measured 1961.8 and 1983.9 respectively. Signal of the dimer (structure shown) resulting from disulfide disproportionation was also detected, expected mass for $C_{172}H_{222}N_{36}O_{22}S_2Na^+$ is 3230.7, measured 3230.6.

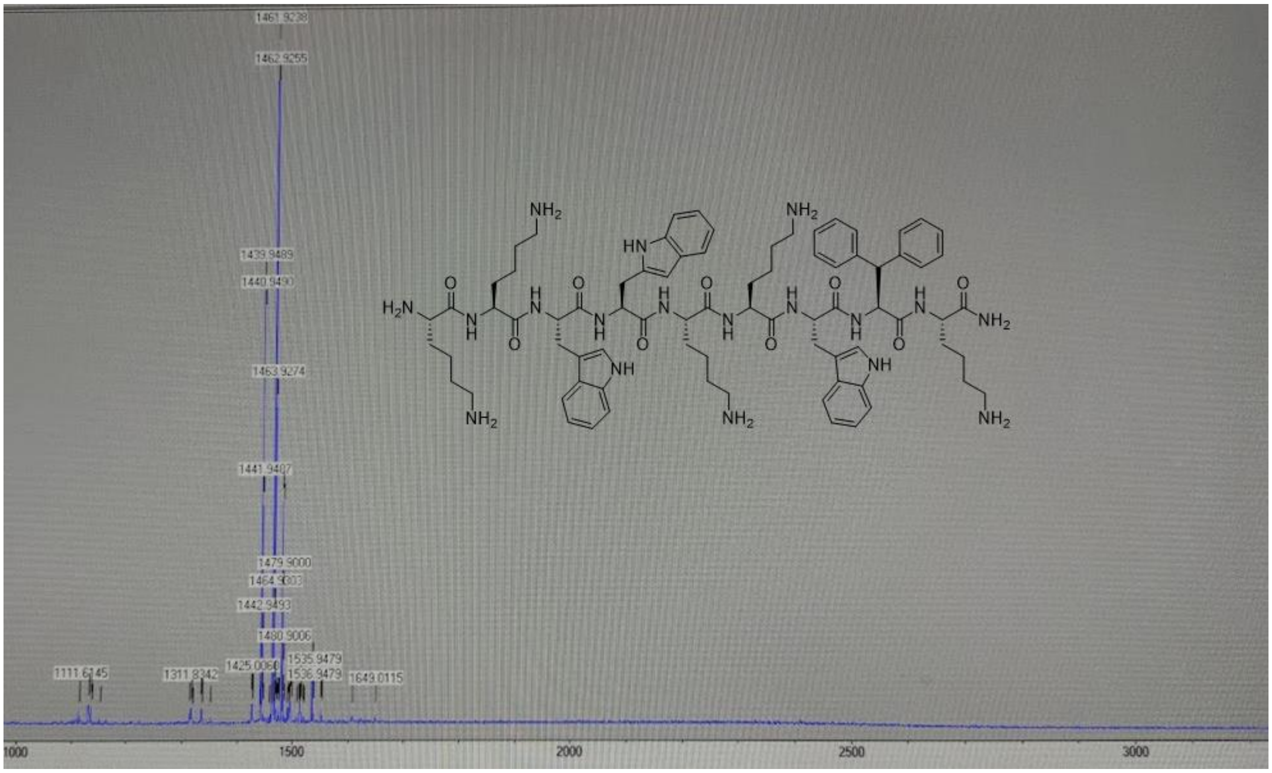

Figure S2.3: MALDI-MS spectrum of the LTX loaded CCPMs after treatment with 5 mM GSH. Expected mass for $C_{78}H_{107}N_{18}O_9^+$ is 1439.8, measured 1439.9 and 1461.9 (Na^+ adduct) respectively.

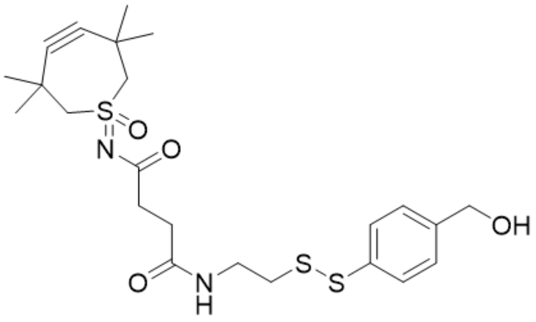

4

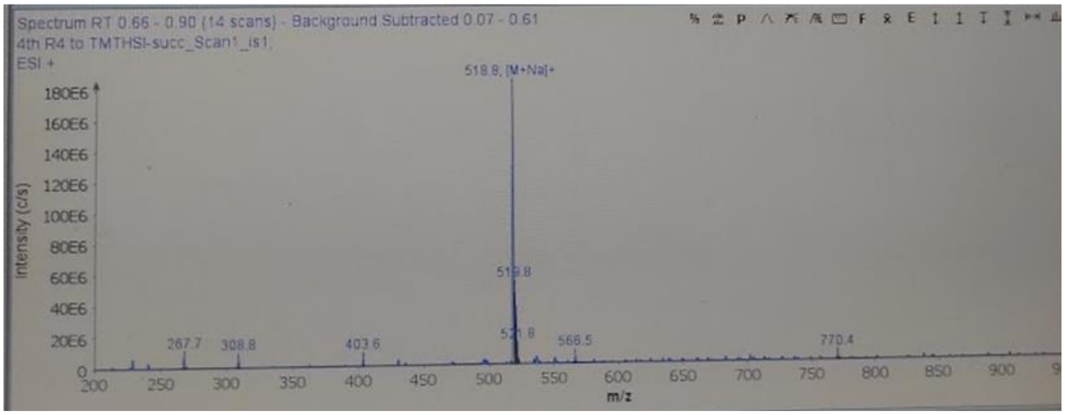

*Figure S2.4: TLC-MS spectrum of compound **4**. Expected mass for $C_{23}H_{32}N_2O_4S_3Na^+$ is 519.1, found 518.8.*

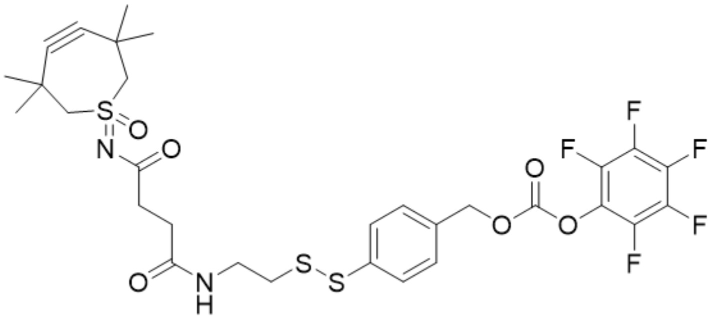

5

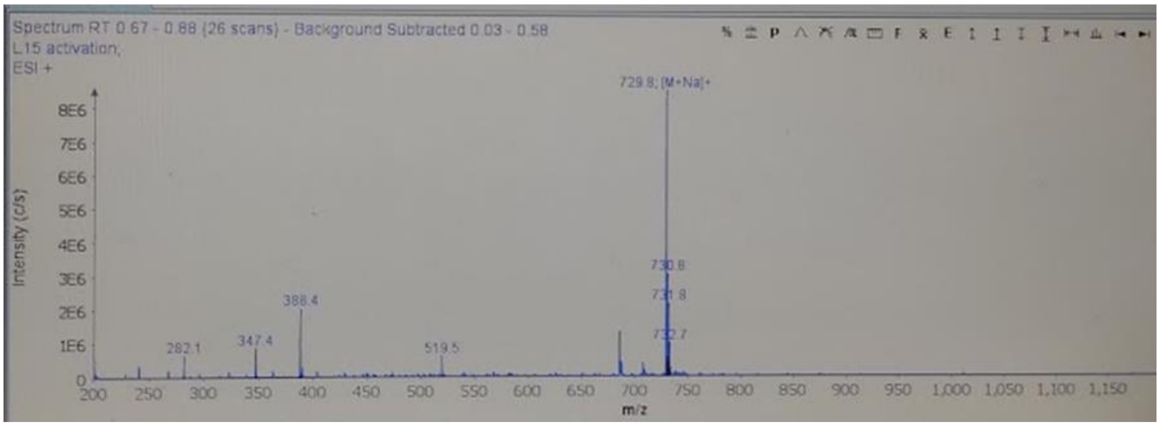

Figure S2.5: TLC-MS spectrum of compound ***5****. Expected mass for* $C_{30}H_{31}N_2O_6S_3F_5Na^+$ *is 729.1, found 729.8*

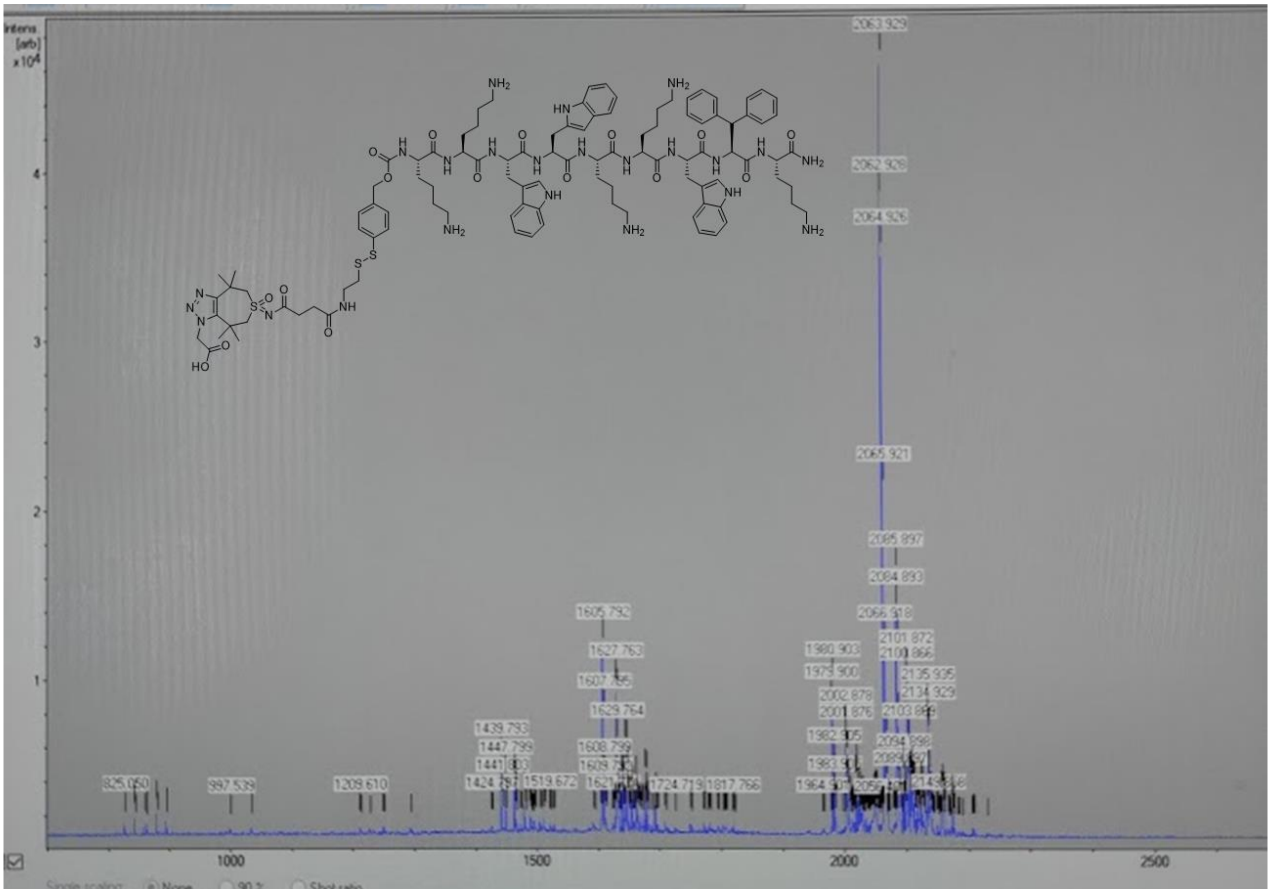

Figure S2.6: MALDI-MS spectrum of a fraction (see figure S3.3, orange chromatogram, R_t = 9.0 mins) that emerged during the click entrapment of linker-LTX into the CCPMs, indicating the presence of uncoupled AAA-linker-LTX. $C_{104}H_{140}N_{23}O_{16}S_3^+$ is 2063.0, measured 2062.9.

3. HPLC Chromatograms

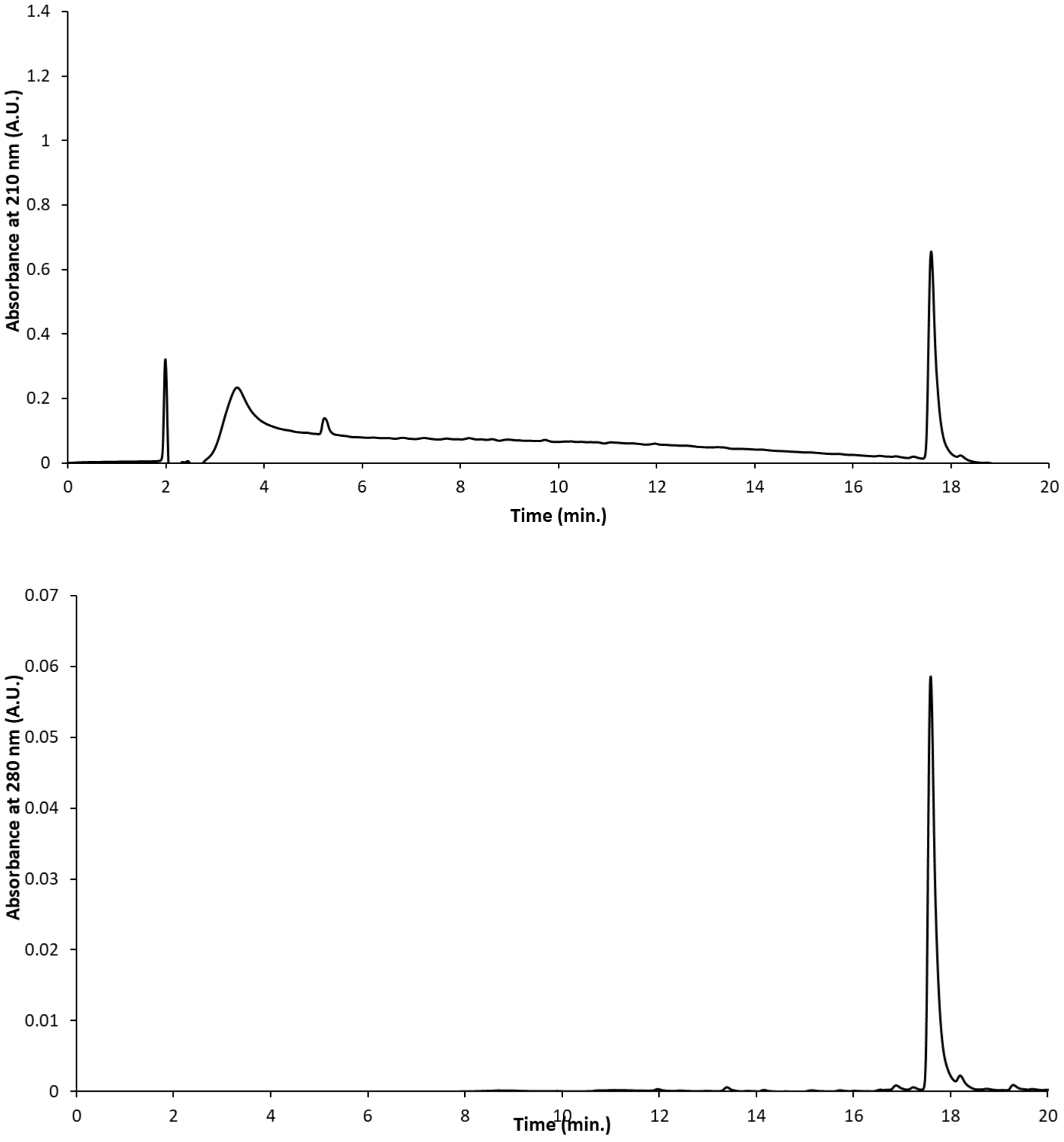

Figure S3.1: HPLC chromatograms of the linker (compound **5***), recorded with 210 nm (top) and 280 nm absorbance (bottom). The linker was dissolved in 50% ACN modified with 0.1% formic acid.*

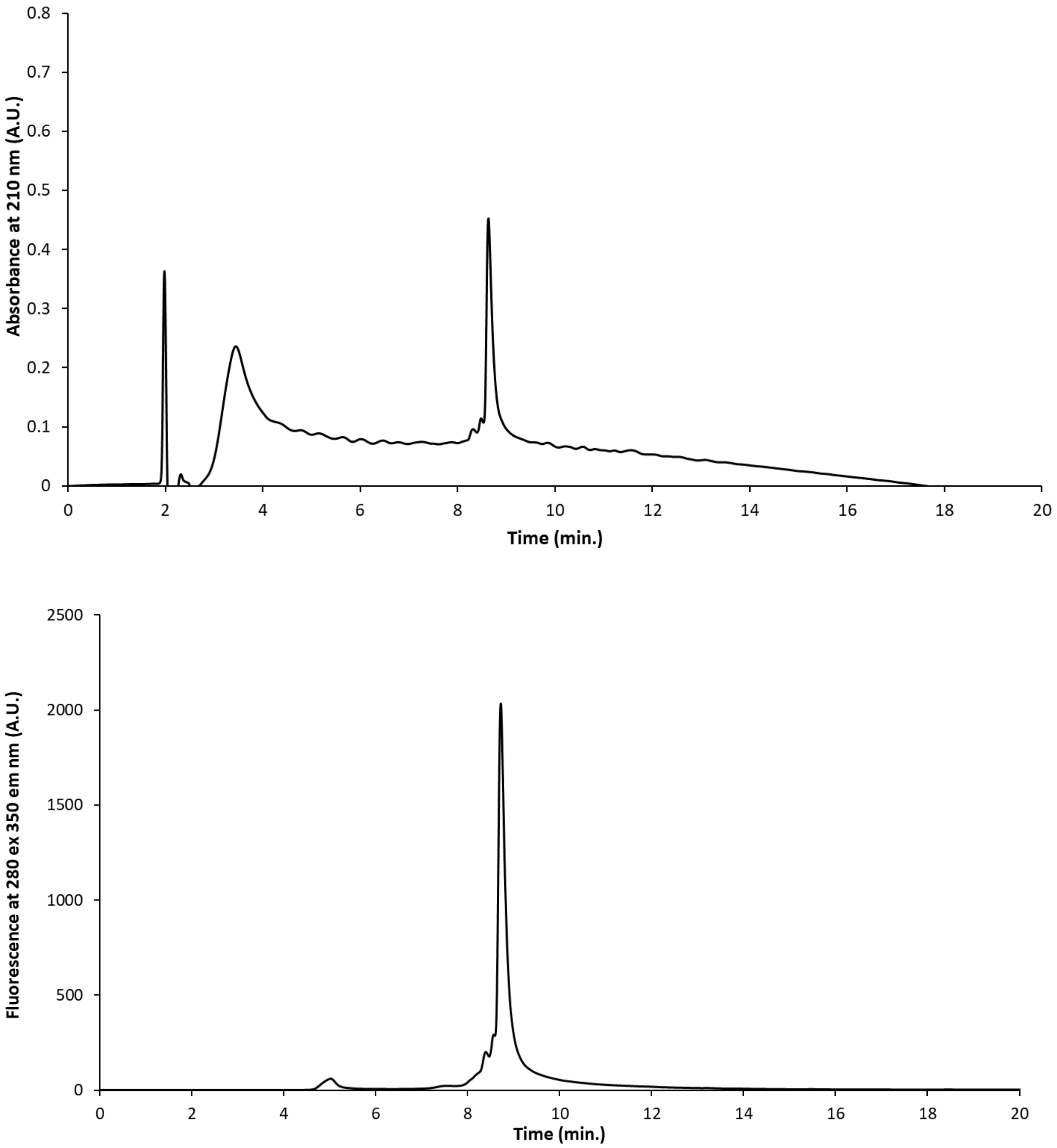

Figure S3.2: HPLC chromatograms of the pure fraction of Linker-LTX, recorded with 210 nm absorbance (top) and $\lambda_{ex}/\lambda_{em}$ 280/350 nm fluorescence (bottom).

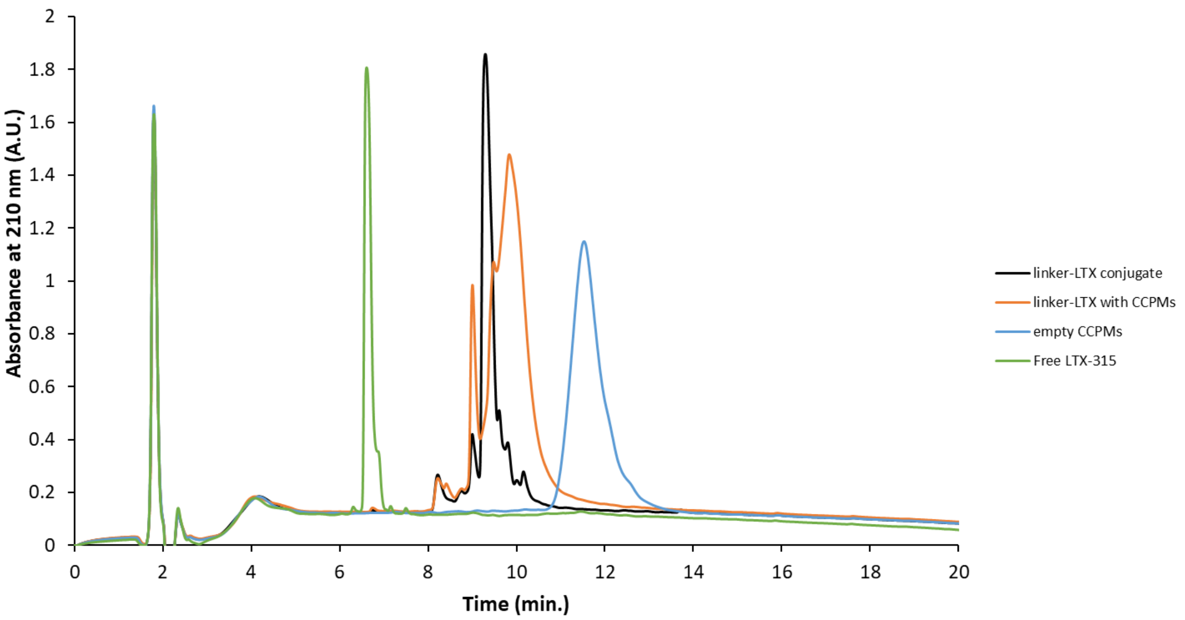

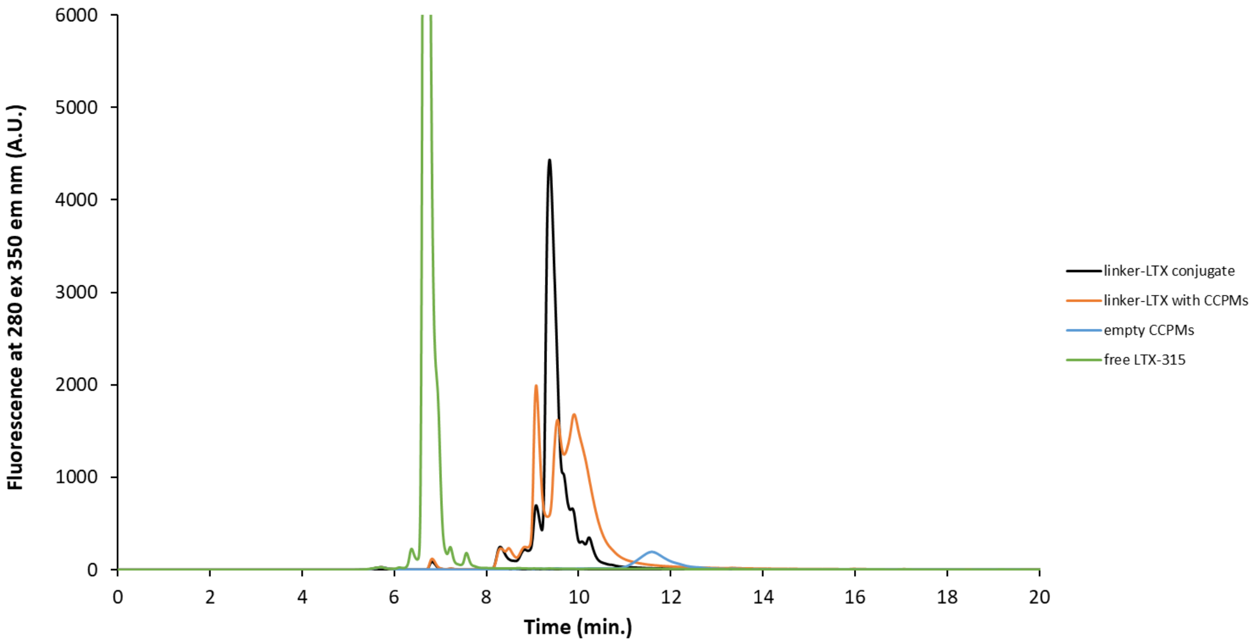

Figure S3.3: HPLC chromatograms of the linker-LTX conjugate (black) incubated with CCPMs for 3 hours without purification (orange), compared to reference empty CCPMs (blue) and free LTX-315 peptide (green), recorded with 210 nm absorbance (top) and $\lambda_{ex}/\lambda_{em}$ 280/350 nm fluorescence (bottom). The peak of linker-LTX (black) disappears upon mixing with CCPMs (orange), and a more hydrophilic CCPM peak as compared to the empty CCPM peak (blue) is observed. No free LTX (green) is detected during the click entrapment. See figure S2.6 for the MALDI spectrum of the newly formed peak at R_t = 9.0 minutes.

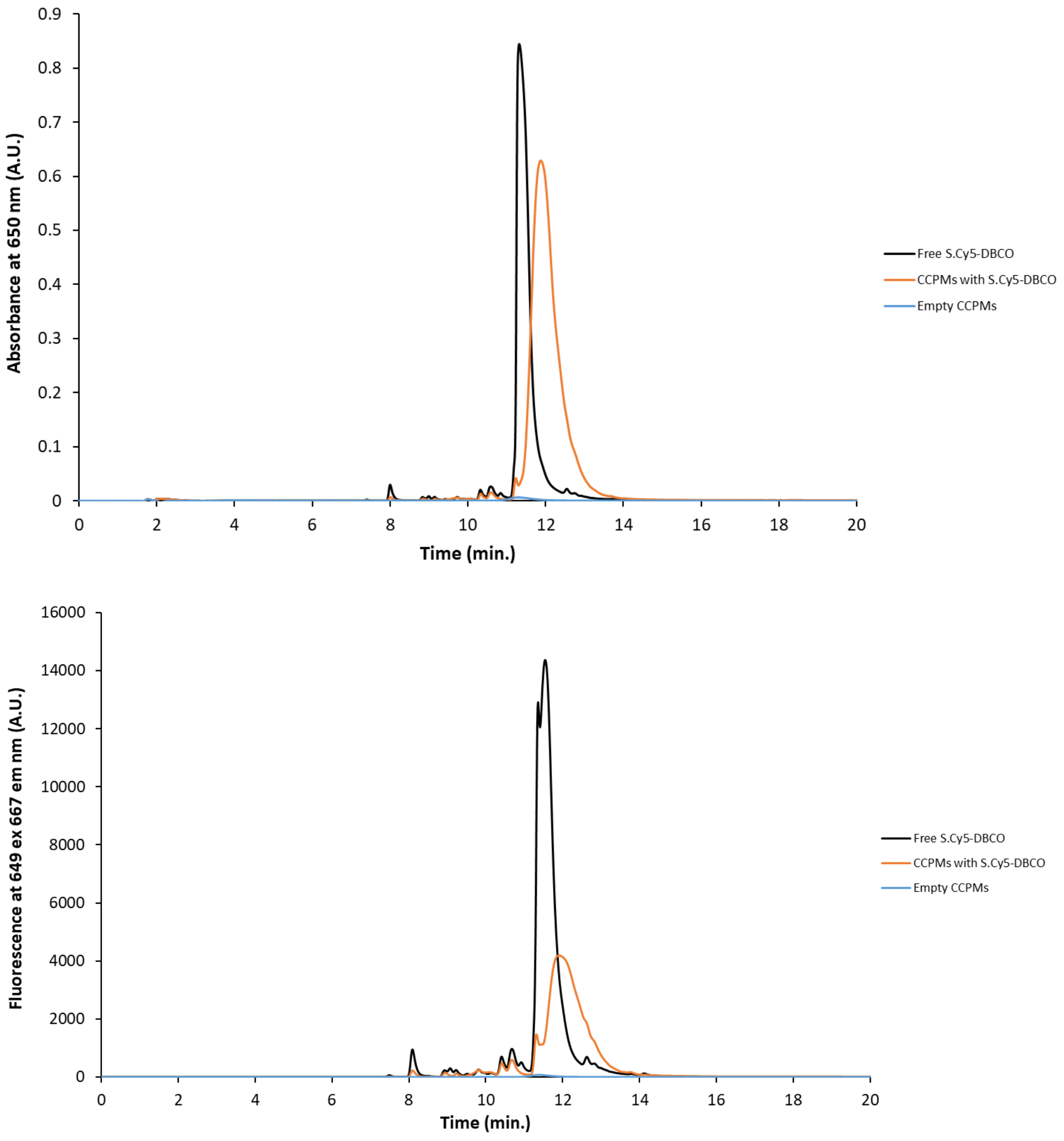

Figure S3.4: HPLC chromatograms of S.Cy5-DBCO (blue) loaded into CCPMs (grey) before TFF purification, compared to reference empty CCPMs (yellow), recorded with 650 nm absorbance (top) and $\lambda_{ex}/\lambda_{em}$ 649/667 nm fluorescence (bottom). See figure S3.5 for the chromatogram after TFF purification.

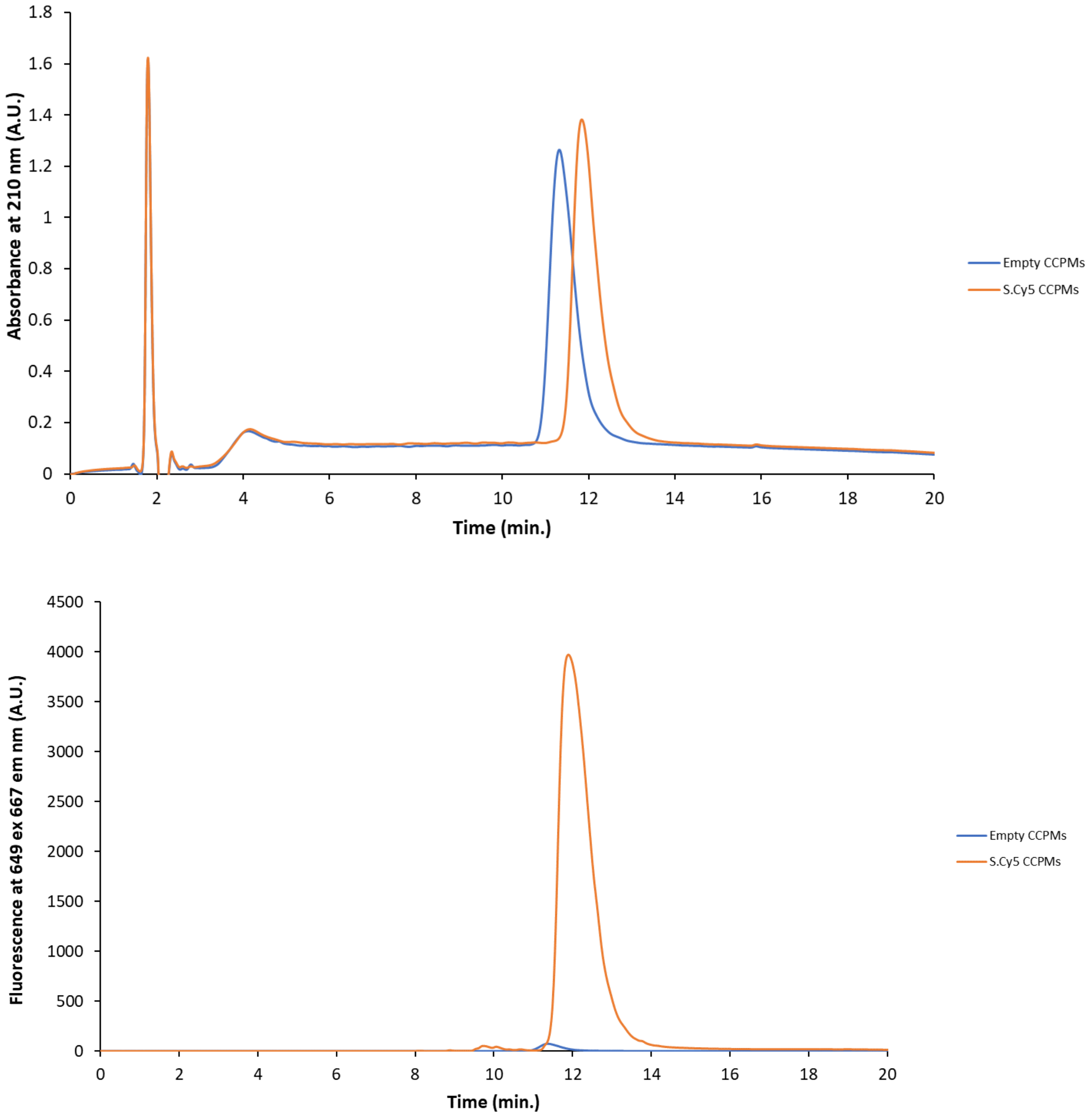

Figure S3.5: HPLC chromatograms of the S.Cy5 loaded CCPMs <u>*after*</u> *TFF purification (orange), compared to reference empty CCPMs (blue), recorded with 210 nm absorbance (top) and $\lambda_{ex}/\lambda_{em}$ 649/667 nm fluorescence (bottom).*

4. UPLC Chromatograms

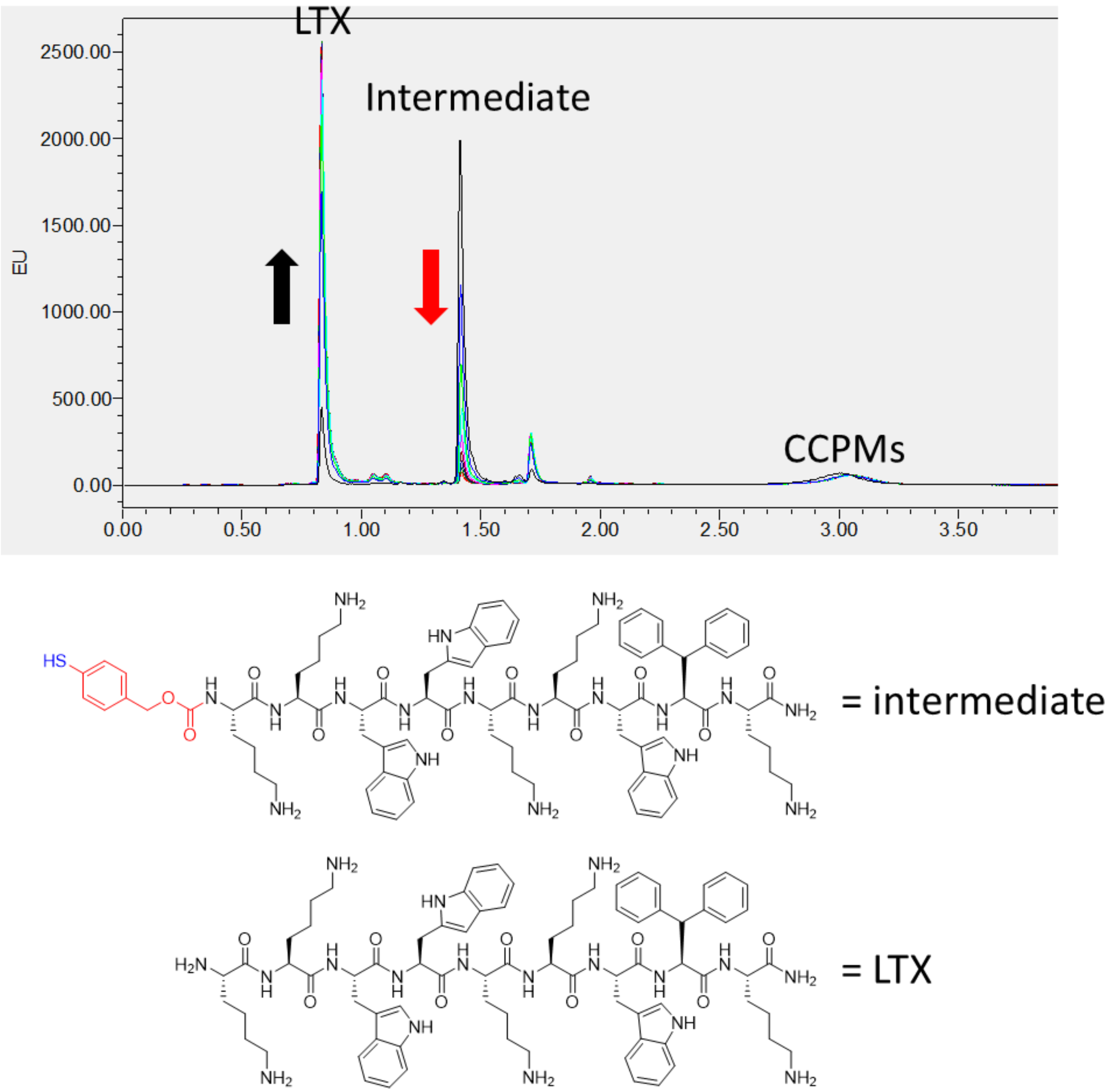

Figure S4.1: Overlayed UPLC chromatograms of the LTX-315 released from the CCPMs under reducing conditions (5 mM GSH), used to quantify the AUC (relative to the highest AUC of native LTX) depicted in figure 1. A clear distinction in retention time is observed between the intermediate state that is almost instantaneously formed and subsequently gradually undergoes 1,6 elimination to form the native LTX peptide. Recorded with $\lambda_{ex}/\lambda_{em}$ 280/350 nm fluorescence.

5. IR spectra

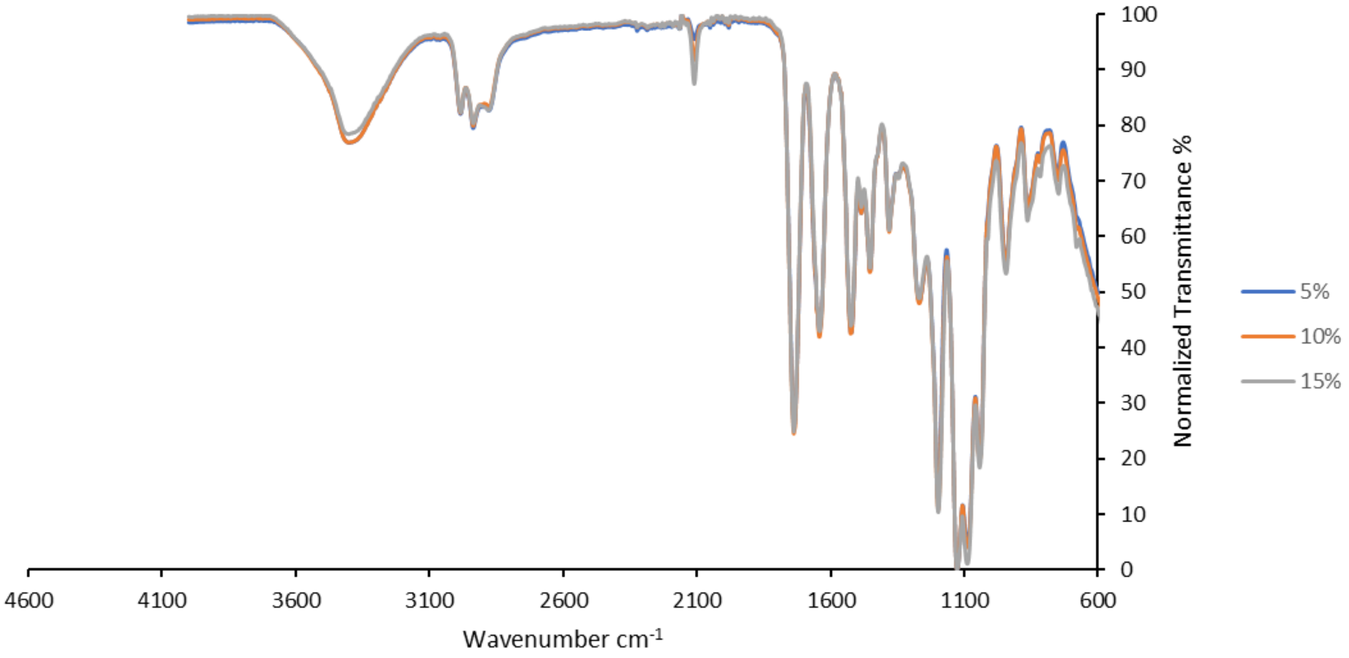

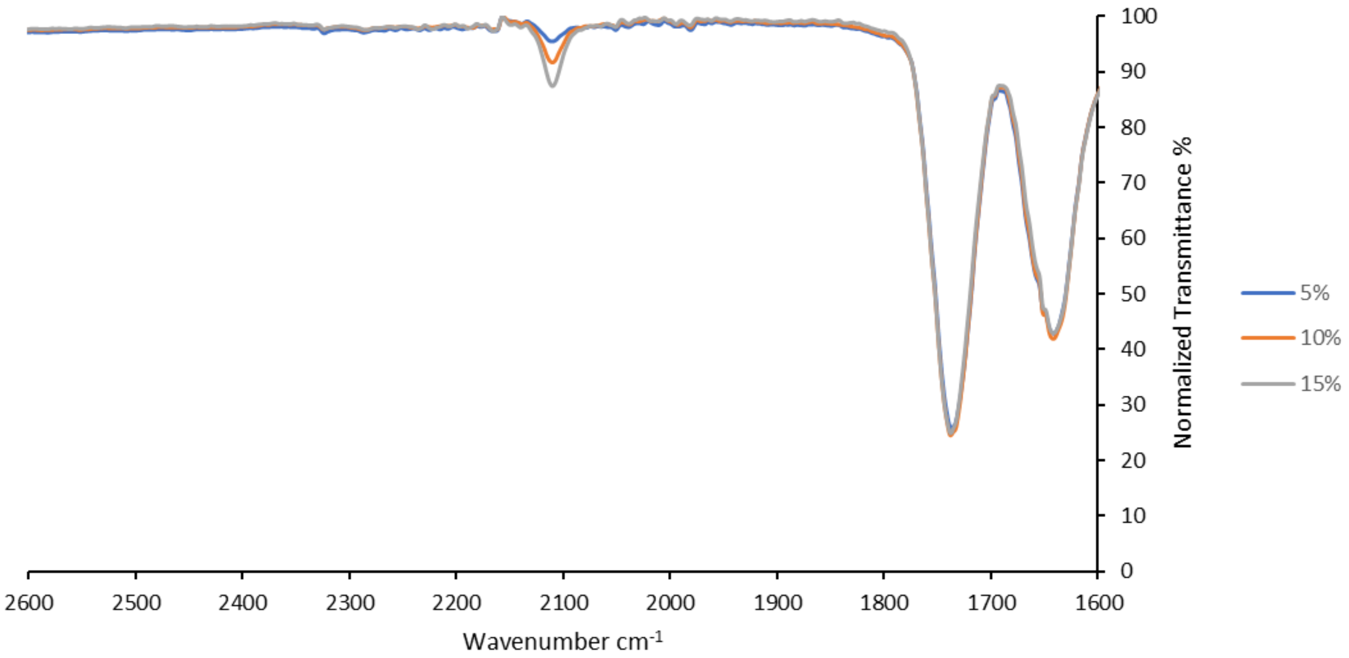

*Figure S5.1: IR spectra of 5, 10 and 15 mol% feed AAA modified polymers (**PA**) showing the characteristic azide peak around 2100 cm^{-1}.*
